# Multisensory Integration Dominates Hypnotisability and Expectations in the Rubber Hand Illusion

**DOI:** 10.3389/fnhum.2022.834492

**Published:** 2022-06-16

**Authors:** Mel Slater, H. Henrik Ehrsson

**Affiliations:** ^1^Event Lab, Department of Clinical Psychology and Psychobiology, University of Barcelona, Barcelona, Spain; ^2^Institute of Neurosciences of the University of Barcelona, Barcelona, Spain; ^3^Department of Neuroscience, Karolinska Institutet, Stockholm, Sweden

**Keywords:** body ownership illusion, rubber hand illusion, RHI, body representation, hypnotisability, imagination, demand characteristics, Bayesian analysis

## Abstract

Some recent papers by P. Lush and colleagues have argued that the rubber hand illusion (RHI), where participants can feel a rubber hand as their own under appropriate multisensory stimulation, may be caused mainly by hypnotic suggestibility and expectations (demand characteristics). These papers rely primarily on a study with 353 participants who took part in a RHI experiment carried out in a classical way with brush stroking. Participants experienced a synchronous condition where the rubber hand was seen to be touched in synchrony with touch felt on their corresponding hidden real hand, or the touches were applied asynchronously as a control. Each participant had a related measure of their hypnotisability on a scale known as the Sussex-Waterloo Scale of Hypnotisability (SWASH). The authors found a correlation between the questionnaire ratings of the RHI in the synchronous condition and the SWASH score. From this, they concluded that the RHI is largely driven by suggestibility and further proposed that suggestibility and expectations may even entirely explain the RHI. Here we examine their claims in a series of extensive new analyses of their data. We find that at every level of SWASH, the synchronous stimulation results in greater levels of the illusion than the asynchronous condition; moreover, proprioceptive drift is greater in the synchronous case at every level of SWASH. Thus, while the level of hypnotisability does modestly influence the subjective reports (higher SWASH is associated with somewhat higher illusion ratings), the major difference between the synchronous and asynchronous stimulation is always present. Furthermore, by including in the model the participants’ expectancy ratings of how strongly they initially believed they would experience the RHI in the two conditions, we show that expectations had a very small effect on the illusion ratings; model comparisons further demonstrate that the multisensory condition is two-to-three-times as dominant as the other factors, with hypnotisability contributing modestly and expectations negligibly. Thus, although the results indicate that trait suggestibility may modulate the RHI, presumably through intersubject variations in top-down factors, the findings also suggest that the primary explanation for the RHI is as a multisensory bodily illusion.

## Introduction

The rubber hand illusion (RHI) (Botvinick and Cohen, 1998) arises from an experimental paradigm at the core of the study of body representation. In the original version of the RHI experiment, the participant sits by a table on which a rubber arm and hand are placed in a position such that they could plausibly be the participant’s own arm and hand. The corresponding real hand is out of sight behind a screen. The experimenter applies tactile stimulation to the participant’s real hand by stroking it and simultaneously applies identical strokes to the rubber hand in the same locations as those applied to the real hand. Hence, the participant sees the rubber hand being stroked and feels the corresponding tactile stimulation on the out-of-sight real hand. The majority of participants will quickly experience a shift in proprioception so that the rubber hand feels as if it were their own and they sense the touches originating directly from the rubber hand (Longo et al., 2008; Reader et al., 2021) with the illusion occurring for most people within 10–15 s approximately (Ehrsson et al., 2004; Lloyd, 2007). If the stroking and tapping on the real and rubber hand are not synchronous, or more precisely, if the degree of asynchrony is greater than approximately 300 ms (Shimada et al., 2009; Ehrsson and Chancel, 2019), then the illusion is not experienced by the majority of participants. Thus, the rubber hand illusion depends on spatiotemporal correspondences of visual and somatosensory signals and is a classic example of a multisensory bodily illusion.

The initial findings of Botvinick and Cohen have stimulated a vast amount of research; according to Google Scholar, the study has had more than 4,400 citations at the time of writing. Part of the reason for the RHI’s popularity is that it is relatively robust, easy to replicate, and flexible to adapt to various experimental settings. It also works well in laboratory exercises for psychology undergraduates, for public demonstrations in popular science events, and in experiments with neurological or neurosurgical patients. Our own experience of public events is that members of the public who are completely naïve to the illusion, typically visibly show their surprise at the moment that the illusion of ownership over the rubber hand occurs. The subjective illusion is often quantified with questionnaires and rating scales (Botvinick and Cohen, 1998; Longo et al., 2008; Reader et al., 2021) in line with a long tradition in psychology of using subjective reports of naïve participants to describe illusions, although psychophysical approaches where illusory perceptions are more rigorously quantified at the individual level are gaining interest (Chancel and Ehrsson, 2020; Chancel et al., 2021b).

In addition to questionnaire rating scales and perceptual judgements, several indirect behavioural and physiological measures for the RHI have been developed. The point of these measures is to provide more objective evidence that the illusion is associated with changes in multisensory body representation, as one would expect from a perceptual bodily illusion. One commonly used such test is the “proprioceptive drift” that registers a change in hand position sense towards the location of the rubber hand during the illusion (Botvinick and Cohen, 1998; Tsakiris and Haggard, 2005). The level of proprioceptive drift is significantly greater after the synchronous condition compared to after the asynchronous and other control conditions; in addition, typically, the stronger the subjective illusion, the stronger is this difference in proprioceptive drift (Sanchez-Vives et al., 2010; Abdulkarim and Ehrsson, 2016). Although proprioceptive drift can occur outside the context of the RHI (Holmes et al., 2006) and the subjective illusion cannot be equated with drift (Rohde et al., 2011), the significant differences in proprioceptive drift between the synchronous and asynchronous conditions have been well replicated (Tsakiris and Haggard, 2005; Tsakiris et al., 2010; Abdulkarim and Ehrsson, 2016; Abdulkarim et al., 2021); proprioceptive drift is related to the RHI because visuoproprioceptive combination and recalibration are key elements of the illusion (Ehrsson et al., 2004; Abdulkarim and Ehrsson, 2016; Fuchs et al., 2016).

Another objective measure is based on the cross-modal congruence task (Pavani et al., 2000), which probes visuotactile interactions related to multisensory body representation. The cross-modal congruence task measures changes in reaction times and relies on the observation that congruent visuotactile stimuli (same fingers) are detected faster than incongruent visuotactile stimuli (different fingers) (Pavani et al., 2000; Zopf et al., 2010). In the RHI implementation of this task, participants respond to spatially congruent or incongruent pairs of visual and tactile stimuli delivered to the hidden real hand (tactile stimuli) and the rubber hand (visual stimuli). The facilitation of the responses for the congruent stimulus pairs during the rubber hand illusion compared to controls (Pavani et al., 2000; Austen et al., 2004; Zopf et al., 2010) provides behavioural evidence that the rubber hand is represented similarly to one’s real hand because the “owned” rubber hand influences the visuotactile spatial interactions in a similar way to a real hand.

An indirect physiological index of the RHI is to register changes in autonomic arousal that occur when a sharp or crushing object (e.g., a knife, syringe, or hammer) is seen threatening the rubber hand; this is called the threat-evoked skin conductance response (SCR) (Armel and Ramachandran, 2003; Petkova and Ehrsson, 2009; Gentile et al., 2013; Fan et al., 2021). Critically, threat-evoked SCR from the illusion condition is compared to a control condition (e.g., asynchronous) because the mere sight of the threat stimuli triggers emotional and surprise reactions that influence the SCR. Notably, during the rubber hand illusion, participants display a stronger threat-evoked SCR in the synchronous than in the control conditions, thereby providing objective physiological evidence that the rubber hand is represented as one’s own hand in terms of emotional defensive processes and reactions (Ehrsson et al., 2007).

In addition to these three classic tests, many other behavioural and physiological tests have been proposed in the literature (Barnsley et al., 2011; Mohan et al., 2012; Maister et al., 2013; Rohde et al., 2013; Butz et al., 2014; De Haan et al., 2017; Kilteni and Ehrsson, 2017). For example, the rubber hand illusion can bias goal-directed pointing behaviour (Kammers et al., 2010; Newport et al., 2010; Heed et al., 2011; Zopf et al., 2011; Fang et al., 2019) and influence sensory attenuation of self-touch (Kilteni and Ehrsson, 2017), which suggests that sensorimotor systems that plan and execute action use information from a multisensory limb representation that has been updated by the illusion.

An equivalent to the RHI has been shown to operate with a virtual hand in virtual reality (Slater et al., 2008), and many of the same results regarding subjective ratings and physiological measures have been found for this “virtual arm illusion”; moreover, effects on goal-directed movements (Burin et al., 2019), pain perception (Matamala-Gomez et al., 2020) and motor cortex activation have been found when the owned virtual hand is threatened (González-Franco et al., 2013). The RHI experimental paradigm has been extended to ownership of a whole body–video of a physical mannequin body seen through a head-mounted display (Petkova and Ehrsson, 2008) and a virtual body (Slater et al., 2010; Banakou et al., 2013). These whole-body versions of the rubber hand illusion follow temporal and spatial rules regarding multisensory stimulation similar to those of the RHI; they are also associated with illusion-specific increases in threat-evoked SCR (Petkova and Ehrsson, 2008; Guterstam et al., 2015) and heart-rate deceleration (Slater et al., 2010). Furthermore, illusions of owning a mannequin or a virtual body can influence cognition and emotion, and such indirect “embodied cognition” effects provide additional indirect behavioural evidence for the basic bodily illusion paradigm. For example, the full-body illusion can lead to changes in implicit racial bias depending on the skin tone of the embodied avatar (Maister et al., 2015), changes in self-concept when experiencing either a friend’s body or a stranger’s body of the opposite sex as one’s own (Tacikowski et al., 2020a,b) and disturbances in episodic memory when body ownership is challenged (Bergouignan et al., 2014; Tacikowski et al., 2020b; Iriye and Ehrsson, 2022).

Finally, the rubber hand illusion is supported by neuroscience. In functional magnetic resonance imaging (fMRI) experiments, the RHI has been associated with increased blood oxygenation level-dependent (BOLD) contrast signals in the premotor cortex and posterior parietal and subcortical regions associated with multisensory integration of body-related sensory signals (Ehrsson et al., 2004; Gentile et al., 2013; Limanowski and Blankenburg, 2016; Grivaz et al., 2017). Moreover, the stronger the activation difference is in the multisensory frontoparietal areas between illusion (synchronous) and control conditions (asynchronous and spatial incongruence), the stronger the illusion as measured with questionnaires (Ehrsson et al., 2004; Ehrsson et al., 2005; Brozzoli et al., 2012; Gentile et al., 2013), proprioceptive drift (Brozzoli et al., 2012), or threat-evoked SCR (Gentile et al., 2013); difference scores on these tests correlate with the condition-specific activations. Electrophysiological recordings of electrical brain activity have shown that the RHI is related to increases in high-gamma activity over frontal and parietal regions (Guterstam et al., 2019) and late evoked and alpha and beta band activity (Rao and Kayser, 2017). Moreover, the RHI and similar hand-ownership illusion are associated with specific changes in functional connectivity and cortical dynamics between frontal and parietal areas, as revealed by electrophysiological (Zeller et al., 2016; Guterstam et al., 2019), neuroimaging (Gentile et al., 2013; Guterstam et al., 2013; Limanowski and Blankenburg, 2015), paired pulse transcranial magnetic stimulation (Karabanov et al., 2017; Isayama et al., 2019), and combined transcranial stimulation and EEG approaches (Casula et al., 2021). Furthermore, single-cell and multiunit recordings from the premotor cortex (Fang et al., 2019) and posterior parietal cortex (Graziano et al., 2000) in non-human primates exposed to versions of the RHI reveal changes in visual receptive field properties, discharge patterns, and local field potentials of multisensory neurons that suggest that the fake hand is represented as the monkey’s own (at least to some degree). These neuroscience observations provide valuable support for the behavioural observations discussed above and provide information about candidate neural mechanisms.

The theoretical understanding of the RHI has concentrated on the multisensory integration of visual, tactile, and proprioceptive information (Tsakiris et al., 2010; Ehrsson, 2012; Blanke et al., 2015; Tsakiris, 2017). Central concepts are the integration of sensory signals from different sensory modalities; the importance of temporal, spatial, and other multisensory congruence rules; and the importance of both bottom-up signals and top-down factors (Kilteni et al., 2015; Samad et al., 2015; Ehrsson and Chancel, 2019; Fang et al., 2019; Chancel et al., 2021a). If you see a hand in a location and orientation such that the hand could be yours, after which you see this hand contingently touched and you feel the touches, based on all your past experiences, there is a very strong likelihood that this is your hand; thus, your brain makes an automatic perceptual decision that the rubber hand is yours, and combines all visual, tactile, proprioceptive, and other body-related sensations into a coherent multisensory experience of the artificial hand as part of your own body, even though you know at the cognitive and conceptual levels that it definitely is not your hand. From a computational perspective, the RHI has been explained as a Bayesian causal inference of a common cause based on probabilistic principles of multisensory perception (Körding et al., 2007). The causal inference model describes how the brain decides whether the visual and somatosensory signals should be integrated (eliciting the illusion) or segregated (no illusion) based on the temporal and spatial correspondences of the multisensory signals and prior knowledge (Kilteni et al., 2015; Samad et al., 2015; Ehrsson and Chancel, 2019; Fang et al., 2019; Chancel et al., 2021a).

However, as with any psychological or perceptual phenomenon, there will still be individual differences between people. Not everyone experiences the RHI, and although this is not unique for a perceptual illusion, the response variables tend to be relatively varied across individuals. In line with the multisensory account, individual differences in the sensitivity in arm position sensing (Horváth et al., 2020) or in the ability to detect whether stimuli presented in two different sensory modalities are synchronous (multisensory simultaneity judgement)—in this case, visuotactile simultaneity judgements (Costantini et al., 2016)—can account for some of the variability across individuals. This makes sense, as participants with more precise proprioception and temporal multisensory perception are likely to be better at detecting the subtle spatial and temporal incongruences that work against the illusion. According to the causal inference model of multisensory perception mentioned above (Körding et al., 2007; Kilteni et al., 2015; Ehrsson and Chancel, 2019), a lower level of proprioceptive precision (reliability) increases the likelihood that the brain infers that a vision of the rubber hand and proprioception have the same cause (Samad et al., 2015; Fang et al., 2019); furthermore, a stronger prior probability for a common cause of vision and somatosensation leads to both poorer visuotactile simultaneity judgements (i.e., a wider temporal window of integration) and a greater tolerance of asynchronies when the RHI is experienced (Chancel et al., 2021a).

Moreover, individual differences at the cognitive level might also influence the illusion experiences of participants through top-down mechanisms and modulate multisensory integration and the evaluation of this experience at a post perceptual metacognitive level. Such individual differences have been previously studied; for example, Haans et al. (2012) examined the impact of individual susceptibility and cognitive distractions and demands, Marotta et al. (2016) investigated sensory suggestibility and found that people with a personality trait more prone to this type of suggestibility were more likely to experience the RHI as reported in the questionnaires, but they did not find a significant relationship to the proprioceptive drift task. Walsh et al. (2015), on the other hand, found a significant correlation between hypnotic suggestibility and proprioceptive drift, but not in the case of the questionnaire ratings. Self-reported psychosis-like characteristics have been reported to influence RHI questionnaire ratings, which suggests that psychosis proneness might enhance reported illusion experiences (Germine et al., 2013; Louzolo et al., 2015). Romano et al. (2021) constructed a new psychometric scale to measure ownership and disownership and found that ownership is correlated with empathy and self-esteem and disownership is correlated with other personality traits. Eshkevari et al. (2012) found that both the subjective experience of the RHI and the associated proprioceptive biases were correlated with eating disorder psychopathology as quantified in self-report measures. Tsakiris et al. (2011) reported how individuals with high levels of the interoceptive ability to count their own heartbeats experienced a weaker RHI, although more recent studies have cast doubt on a relationship between RHI and cardiac interoception (Crucianelli et al., 2018; Horváth et al., 2020; Critchley et al., 2021). Collectively, the literature on interindividual differences suggests that variations in perceptual, emotional and cognitive processing between individuals can modulate the rubber hand illusion at different levels, although many questions remain unanswered.

However, a recent study that investigated individual differences in the RHI went much further in regard to its conclusion (Lush et al., 2020). These authors found a correlation between hypnotic suggestibility and illusion questionnaire ratings (*R*^2^ = 0.09) in the synchronous condition and a weaker relationship with proprioceptive drift (*R*^2^ = 0.02). Similar relationships were observed between hypnotic suggestibility and the illusion ratings in the asynchronous condition (*R*^2^ = 0.09). Lush and colleagues did not simply conclude that trait hypnotic suggestibility may modulate the RHI in line with previous studies but went much further and suggested that the RHI may be *entirely* explained by hypnotic suggestibility, expectations, and demand characteristics; they also suggested that multisensory mechanisms may only play a minor role, if any. A commentary (Ehrsson et al., 2022) argued that these conclusion were too strong given that no reliable relationship was found when the asynchronous control condition was used in the analysis and that the illusion strength was defined as the difference between the illusion condition and the control condition. However, Lush and colleagues disagreed with this objection and maintained that the most straightforward interpretation is that the RHI is caused by a combination of suggestibility, expectations, and demand characteristics (Seth et al., 2021; Lush and Seth, 2022). The original article by Lush et al. (2020) has attracted much interest in the community and beyond and has quickly become a highly cited work. Lush and colleagues’ strong claims are fascinating, as they seem to raise fundamental questions about the relationship between cognition and perception; to non-experts, these claims bear superficial similarities with the replication crises in psychological research where famous psychological effects have turned out to be unsupported upon closer examination (Makin, 2020), although the RHI is not difficult to replicate at all and Lush et al. (2020) replicated it. However, are Lush and colleagues’ strong conclusion supported by the data, and are they reasonable, given the previous literature?

To address this question, we conducted a reanalysis of Lush’s publicly available data (Lush et al., 2020). We have found that while what was reported was not incorrect in itself, the results presented were partial. For example, only correlations associated with linear regressions were reported (effectively the slope of the regression lines) but not the intercepts, which in this case carry particularly important information. Moreover, Lush et al. (2020) did not address the statistical assumptions underlying their analysis, which is an important omission given the radical nature of their claims in comparison to past findings. Therefore, we were particularly interested in characterising the relationship between trait hypnotic suggestibility and the RHI illusion measured in the two conditions in detail and in examining whether these relationships change or remain constant for increasing levels of hypnotic suggestibility. We also have investigated the relative contribution of multisensory integration, hypnotic suggestibility, and expectation effects to determine which factor(s) are dominant.

To this end, in the following we consider the underlying assumptions of normality of the residual errors of their regression analyses; furthermore, we consider not only correlations but also intercepts, which in this case provide important additional information. Moreover, we explicitly consider the ranges of the response variables and the residual errors of the regression model fits and influential points, which may otherwise bias the results. We show that the regression analyses result in different conclusion when the intercepts are taken into account. In particular, we show that the RHI questionnaire scores, although correlated with hypnotisability, show a stronger illusion for the synchronous than for the asynchronous at each level of hypnotisability. We show this through a simple graphical presentation of the raw data and in a Bayesian statistical model that takes into account the valid ranges of the response variables and that does not suffer from outliers or influential points. Furthermore, we model proprioceptive drift in a way that takes into account both symmetric departures from the mean drift and the added influence of synchronous compared to asynchronous stimulation. Finally, we examine the potential contributions of expectations and show that their impact on the subjective RHI ratings is small, even smaller than that of hypnotisability. Model comparisons further show that the contribution of multisensory conditions dominates two- to threefold over both expectations and hypnotisability, which suggests that the main explanation for the results is the multisensory bodily illusion. Collectively, our findings are in line with the traditional view that multisensory integration is the major causal factor for the RHI, and we find that by relying solely on correlations, Lush et al. (2020) were not able to find the effect of multisensory integration.

## Materials and Methods

This paper relies wholly on data supplied by Lush et al. (2020), and detailed methods are described therein. An “opportunity sample” consisting of 353 undergraduate students taking part in a psychology laboratory practical session (78% females) was recruited. First, and crucially for the present paper, all participants took part in a hypnotisability screening procedure. To this end, each participant was scored for hypnotisability using the Sussex-Waterloo Scale of Hypnotisability (SWASH) (Lush et al., 2018). In particular, the subjective score scale used in the analysis ranges from 0 to 5, with a score of 5 indicating a greater level of hypnotisability. We refer to the variable in the analysis as *swash* and the scale itself as SWASH.

Next, the participants received pre-recorded information about the RHI via headphones. The participants were explicitly told how the RHI illusion works and that ‘‘the combination of information from touch and vision induces an illusory experience of ownership over the rubber hand.’’^1^ The participants were further divided into three groups that received different instructions about the synchronous and asynchronous conditions (*instruction conditions*): 114 received instructions that the RHI effect would be stronger in the synchronous condition, 115 were told that the effect would be strongest in the asynchronous condition, and 124 were given no instruction about which of the two conditions should elicit the illusion. The participants then had to rate their expectancies of whether they thought they would experience the rubber hand as their own in each of the two conditions (see further below). Lush and colleagues’ hypothesis was that the RHI expectancy ratings would differ across conditions depending on the type of instruction, a result that was not supported by the data; therefore, all the data were pooled and analysed as a single sample. These data (*n* = 353) were used in the current analyses.

The participants were then tested on the RHI using their right hand and a rubber right hand. They each experienced both the synchronous and asynchronous conditions of the rubber hand experiment in counterbalanced order. The participants sat in front of a table, and one of 16 different experimenters sat on the opposite side. The rubber hand was placed in front of the participant inside a box, and the participant could see the fake hand through a square window on top of the box. The participant’s real hand was kept still inside the box in a visually occluded location 20 cm to the right of the rubber hand. The experimenter used a paintbrush to stroke the rubber hand’s middle finger at approximately 1 Hz for 60 s while simultaneously stroking the middle finger of the participant’s real hand with an identical brush either synchronously or asynchronously.

The RHI was measured following usual practice with a questionnaire and a proprioceptive drift test. There were three crucial questionnaire statements scored on a Likert scale ranging from –3 (strongly disagree) to 3 (strongly disagree):

*s1*: It seemed as if I were feeling the touch of the paintbrush in the location where I saw the rubber hand touched.
*s2*: It seemed as though the touch I felt was caused by the paintbrush touching the rubber hand.
*s3*: I felt as if the rubber hand was my hand.

These questions were administered after each of the synchronous and asynchronous exposures. The three question scores were combined into their average to obtain an “RHI score”; hence, there were two variables as follows:

*ss* = (*s*1 + *s*2 + *s*3)/3 (synchronous)

*sa* = (*s*1 + *s*2 + *s*3)/3 (asynchronous)

The study also included a fourth control statement (*s4*), but we did not use the ratings from this statement in the current analyses since the illusion statements are more informative for the current research questions and only this single control statement was included (typically RHI studies use at least 3–6).

In addition, participants were asked to point without visual feedback towards where they felt their (real) right hand to be both before and after the rubber hand procedure. The window on top of the box was closed so that the participants could no longer see the rubber hand, and a ruler was placed on top of the box. The participants were asked to indicate the point on the ruler where they thought the index finger of their right hand was located inside the box, and the position was noted by the researcher. The proprioceptive drift is the difference between this indicated real right index finger location after the illusion induction and the location before. We denote these differences by *dpdsync* for the synchronous condition (*d*ifference in *p*roprioceptive *d*rift for the *sync*hronous condition) and *dpdasync* for the asynchronous condition.

The typical RHI findings are that *mean*(*ss*) is high (typically above 1 on average) for the synchronous condition, while *mean*(*sa*) is significantly lower (typically below 0) for the asynchronous condition. Critically, *mean*(*dpdsync*) > *mean*(*dpdsync*) is taken as proprioceptive drift evidence of a successful RHI induction.

In the Section “Results,” we first conduct a normal linear regression in line with the correlational analysis by Lush et al. (2020), and we show that by only reporting the correlations between *swash* and the RHI response variables and not the regression line intercepts, a critical aspect of the results was missing from the original article. Next, we present the data descriptively and show through a graphical analysis that the distributions of the questionnaire scores differ substantially between the asynchronous and synchronous conditions independently of the *swash* scores. Following this, we continue with a descriptive analysis of the proprioceptive drift results and again show a difference between the asynchronous and synchronous conditions independently of *swash*. In these descriptive analyses, we are only reporting the actual data and not attempting statistical inference. Where significance values are presented, these are only to illustrate the strength of a correlation or difference for purely descriptive purposes.

For statistical inference, we present a Bayesian statistical model that brings all the analyses together and demonstrates major differences between the asynchronous and synchronous responses independently of *swash*. We use this model to generate new pseudorandom data and compare these with the original data, showing that the model predicts the results well. Whether we use normal linear regression, descriptive analysis or the Bayesian model, we find that the synchronous condition results in stronger illusion scores than the asynchronous condition for the questionnaires and that the proprioceptive drift is greater for synchronous than asynchronous conditions. Although *swash* is positively correlated with the RHI questionnaire score, the synchronous scores are greater overall than the asynchronous scores, indicating the major role of multisensory integration, with hypnotisability as a secondary modulating individual difference factor. Finally, we compare different models and examine the relative importance of hypnotisability and expectations (“expectancy ratings” obtained before the RHI experiments, as mentioned above and further described below) and show that the contribution of the multisensory conditions dominates.

The statistical software used in our analysis was Stata 16 (stata.com) for the descriptive analysis and rstan,^2^ the R interface to Stan (see below) for the Bayesian analysis. For convenience, the details of the different analyses and their rationale are described in the respective Section “Results” below.

## Results

### Normal Linear Regression for the Questionnaire Scores

As described above, using the pooled *n* = 353 observations for the RHI, we first carried out a normal linear regression analysis to examine the influence of *swash* on the RHI response variables using the same approach as that used by Lush and colleagues. We replicated their basic correlation findings in the case of *ss* on *swash R*^2^ = 0.08 (*P* < 0.0005) and in the case of *sa* on *swash R*^2^ = 0.09 (*P* < 0.0005).^3^ Based on their interpretation of these results, Lush et al. (2020) stated that hypnotisability “predicts” the RHI.

However, Lush et al. (2020) did not report an important aspect of the results of the regression of the questionnaire scores (*ss* and *sa*) on *swash*. For *ss*, we found that the slope is 0.58 (*t* = 5.52, *P* < 0.0005, 95% confidence interval 0.37 to 0.79), and for *sa*, we found that the slope is 0.68 (*t* = 6.01, *P* < 0.0005, 95% confidence interval 0.46 to 0.91), and these slopes were emphasised by Lush and colleagues. This leads to the conclusion that *ss* and *sa* are positively associated with *swash*, as reported by Lush et al. (2020). However, it is also important to consider the intercepts of the corresponding regression lines. For *ss*, we find that the intercept is 0.33 (*t* = 1.75, *P* = 0.08, 95% confidence interval –0.04 to 0.70). For *sa* it is –1.47 (*t* = –7.25, *P* < 0.0005, 95% confidence interval –1.87 to –1.07). From this, we can conclude that even though there may be a positive association between the RHI questionnaire score and *swash*, this corresponds to two almost parallel lines with a positive slope, but the line for *ss* is above that of *sa* (as the intercept of *sa* is clearly negative, but the intercept for *ss* is not). What this means is that even though *swash* has an effect, as reported by Lush et al. (2020), at every level of *swash*, the synchronous score is greater than the asynchronous score.

**FIGURE 1:**
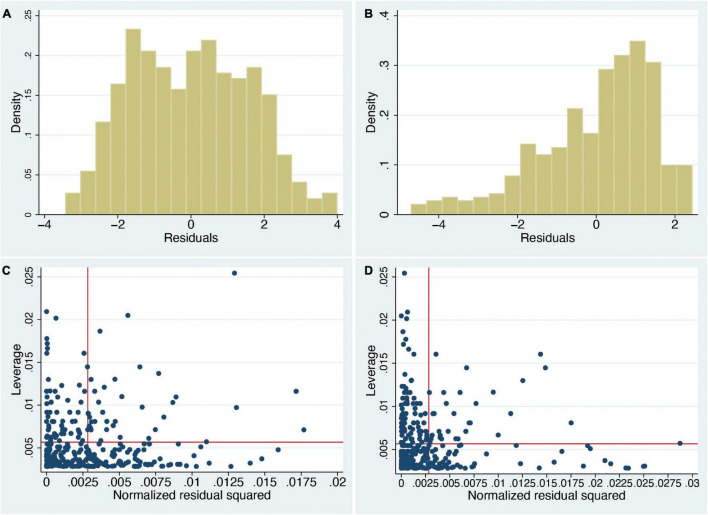
Histograms of residual errors of the regression fits and leverage plots of the normal linear regression analyses (*n* = 353). **(A)** *sa* on *swash*, **(B)** *ss* on *swash*, **(C)** leverage plot of *sa* on *swash*, and **(D)** leverage plot of *ss* on *swash*. *sa* and *ss* denote the RHI questionnaire scores in the asynchronous (*sa*) and synchronous (*ss*) conditions, respectively. Density (y-axis) is the empirical probability density so that areas under the histogram correspond to probabilities. Residual errors are the differences between the fitted and observed values (x axis). Note that if *swash* would explain all variance in the RHI ratings, then the residuals in panels **(A,B)** should be similar and centred around 0, but as can be seen, there are clear differences with ratings tending to higher values in *ss* **(B)**. Panels **(C,D)** show the normalised squared residuals (x axis) against the leverage values (y axis). The vertical line shows the mean of the normalised squared residuals and the horizontal line the mean leverage. High leverage values are especially important, since they indicate that the corresponding data points are very influential in the sense that they strongly influence the regression fit, whereas high residual errors show points that are far from the regression line. It can be seen that there are some very high leverage values, more than 2 or even 3 times the mean leverage, and likewise some very high residual errors, more than 2 or 3 times their mean.

However, there are some important considerations to be taken into account with this type of analysis. The first is that the normality assumption is violated. The residual errors of a regression fit should be approximately normally distributed. Figures 1A,B show the distributions of the residual errors, which in both cases are clearly far from normal (In case of interest, the Shapiro–Wilk test rejects the null hypothesis of normality, in both cases with *P* < 0.0003).

Second, there are several influential points that may influence the regression line, which is an important consideration in linear regression analyses. This can be shown by standard leverage against normalised squared residual error plots (Figures 1C,D). Technically, a leverage value for an individual point is the partial derivative (rate of change) of the fitted value of a response variable with respect to the observed value. Hence, a large leverage value means that a small change in the observed value would result in a large change in the fitted value, indicating that the individual response may distort the model fit (Nurunnabi et al., 2016). While there are no absolute criteria as to what are considered as high leverage values, it can be seen that there are some values that are more than two or even three times the mean, and similarly for the squared residuals. Points far above the horizontal line have excessive leverage values, and points far to the right of the vertical line have large residual errors. We would want there to be no such influential or outlying points, but there are clearly many. An influential point can greatly affect the result of a regression, whereas a large residual error indicates a poor regression fit to the corresponding data points.

Third, the statistical model is not appropriate to the range of values of the response variables. The *ss* and *sa* scores are bound between –3 and 3, yet there is nothing in the model that takes into account these constraints; this means that it might be possible for fitted values to be outside of this range (although this does not happen in these cases). In fact, the ranges of fitted values are squashed compared to the ranges of *ss* and *sa*, which are both from –3 to 3. The fitted values for *ss* range from 0.33 to 2.50, and the range for *sa* is from –1.47 to 1.08. Especially in the case of *ss*, there is quite a difference between the range of observed and fitted values.

Figures 1A,B have another important implication. They show the distributions of questionnaire scores after having eliminated the linear effect of *swash*; i.e., they are the residual errors of the regression. We note that after eliminating the linear influence of *swash*, there remains an observable difference between the two distributions. The weight of the synchronous distribution is clearly shifted towards higher affirmative scores (Figure 1B), and the asynchronous distribution is essentially symmetric with a broad and somewhat even distribution around approximately 0 (Figure 1A). However, if *swash* were the totally dominating factor driving the RHI questionnaire scores, we would expect that what would be left over after eliminating it would just be noise. There is, though, a systematic difference between the asynchronous and synchronous conditions in line with the explanation that the illusion ratings are strongly driven by different patterns of multisensory stimulation.

### Distributions of the Questionnaire Scores

**FIGURE 2:**
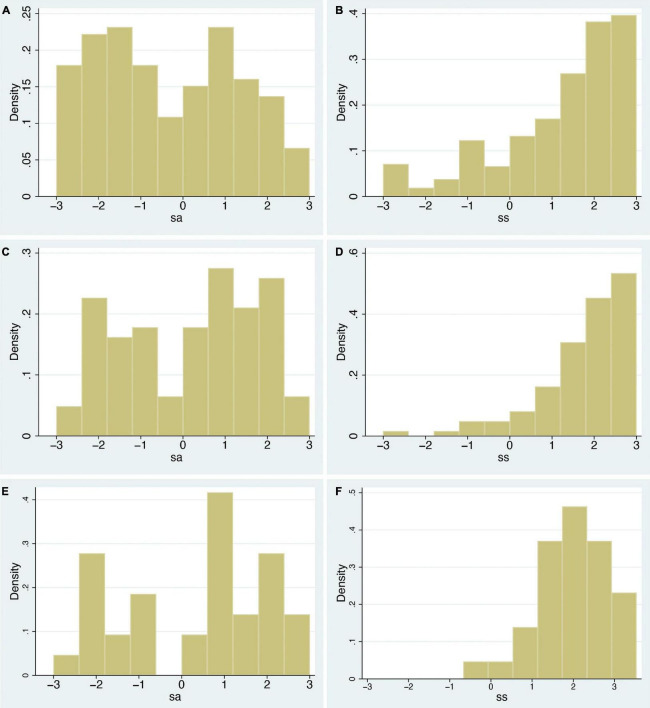
Histograms of the *sa* and *ss* variables for varying levels of *swash*. **(A,B)** For all 353 individuals, **(C,D)** for those with *swash* > 2 (*n* = 103), and **(E,F)** for those with *swash* > 2.64 (*n* = 36). The x-axis shows the ordinal scale of the seven-point scale for the questionnaire responses (from –3 to +3), and the y-axis shows the empirical probability density.

A correlation is a single statistic that summarises the degree of linear relationship between two variables, yet it may not well represent the full relationship between them, especially when underlying distributions are not considered. In this section, just by examining the raw data, a quite different picture emerges compared to only considering correlations. If there was a dominating effect of *swash* on how people responded to the RHI, we would expect that (i) the distributions of *ss* and *sa* would be similar since the multisensory integration aspect (whether the stimulation were synchronous or asynchronous) would have little effect; more importantly, we would expect that (ii) for greater values of *swash*, the effect of multisensory integration should be overridden by the hypnotisability effect. The 70th and 90th percentiles of *swash* are 2.00 and 2.64, respectively. Figures 2A,B show the distributions of *ss* and *sa* across all *n* = 353 participants. Figures 2C,D show these distributions for those with *swash* > 2.00 (*n* = 103). Figures 2E,F show those with *swash* > 2.64 (*n* = 36).

As can be seen in Figure 2, the distributions for asynchronous and synchronous conditions are quite different in all cases. For the synchronous cases, the weights of the distributions are towards higher levels of *ss*, and this hardly varies for increasing levels of *swash*. For the asynchronous cases, the distributions are essentially uniform. In case of interest, the Kolmogorov–Smirnov test rejects the null hypotheses that the asynchronous and synchronous samples are from the same distribution in each case (*P* = 2.2×10^−16^, 9.7×10^−10^, 8.8×10^−5^, for the three cases, respectively).

To further examine the possible impact of *swash* on the questionnaire scores, we plotted the proportions of relatively high scores (*sa*, *ss* ≥ 1) and high scores (*sa*, *ss* ≥ 2) for each value of *swash* from 0 to 2.5 in steps of 0.1 (It is not possible to go much higher than 2.5 because *n* decreases too much). Let *prop*_*sa* ≥ 1_(*sw*) be the proportion of observations with *sa* ≥ 1 conditional on *swash* > *sw*, and similarly for *prop*_*ss* ≥ 1_(*sw*) for the synchronous case (*swash* is the variable name and *sw* the particular value). Similarly, for *prop*_*sa* ≥ 2_(*sw*), *prop*_*ss* ≥ 2_(*sw*) for scores ≥ 2. We computed the pairs resulting in 26 coordinates:


(1) $$\begin{array}{c} \left(prop_{sa\geq s}\left(sw\right),prop_{ss\geq s}\left(sw\right)\right)\\ sw=0,0.1,0.2,\ldots ,2.5;\\ s=1,2 \end{array}$$

**FIGURE 3:**
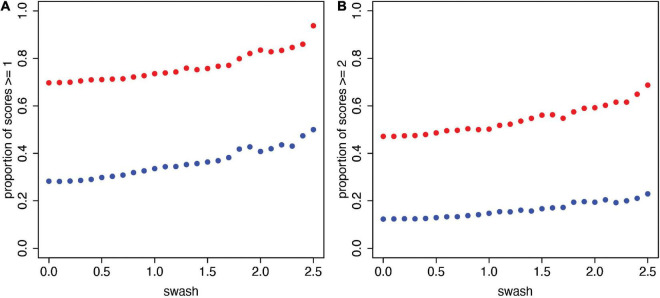
The proportions of relatively high RHI scores (*sa*, *ss*) for increasing values of *swash* (*n* = 353). **(A)** Proportion of scores ≥ 1. **(B)** Proportion of scores ≥ 2. These are the scatter plots of (*sw*, *prop*_*sa* ≥ *s*_(*sw*)) (asynchronous, blue) and (*sw*, *prop*_*ss* ≥ *s*_(*sw*)) (synchronous, red) for *sw* = 0 to 2.5 in steps of 0.1 and for each of *s* = 1 **(A)** and *s* = 2 **(B)**. It can be seen that there is a stable relationship, with higher proportions of participants reporting a stronger RHI in the synchronous condition (*ss*) compared to the asynchronous condition (*sa*) across the range of trait hypnotisability scores (*swash*) possible with these data.

Figure 3A shows the scatter plots of these values in the case of *sa*, *ss* ≥ 1. It is clear that in these data, there is a very strong and essentially constant relationship, with the synchronous case being substantially greater independent of the level of *swash*. Similarly, Figure 3B shows the plots for *sa*, *ss* ≥ 2. Of course, in this case, the proportions are lower, but the same relationship holds; at every level, the synchronous proportions are higher than the asynchronous proportions in these data. Although the level of hypnotisability increases the likelihood of higher illusion scores (in line with the reported correlations and previous work), the dominant factor in these data is whether the stimulation is synchronous or asynchronous. In other words, participants who are more hypnotisable are somewhat more likely to give higher scores, but multisensory integration maintains a difference between the asynchronous and synchronous conditions. We have presented these results as they are in the underlying data without any statistical inference. We turn to that in Section “A Statistical Model for the Questionnaire Data.”

### Normal Linear Regression for Proprioceptive Drift

Recall that for proprioceptive drift, there are two response variables: *dpdsync* for synchronous drift and *dpdasync* for asynchronous drift. For *dpdsync* (proprioceptive drift in the synchronous condition), we observed *R*^2^ = 0.02 (*P* = 0.011) and for *dpdasyncR*^2^ = 0.00 (*P* = 0.45), which again replicates Lush and colleagues’ findings. Moreover, since the regression indicates that the slope for the synchronous case is positive (=0.54, *t* = 2.54, *P* = 0.01, 95% confidence interval 0.12 to 0.96) but the slope for the asynchronous case is not (0.15, *t* = 0.76, *P* = 0.44, 95% confidence interval –0.23 to 0.53), and the intercepts are not significantly different from 0 in both cases, again this means that for every level of *swash*, the regression lines indicate that the drift for the synchronous case is greater than that for the asynchronous case.

**FIGURE 4:**
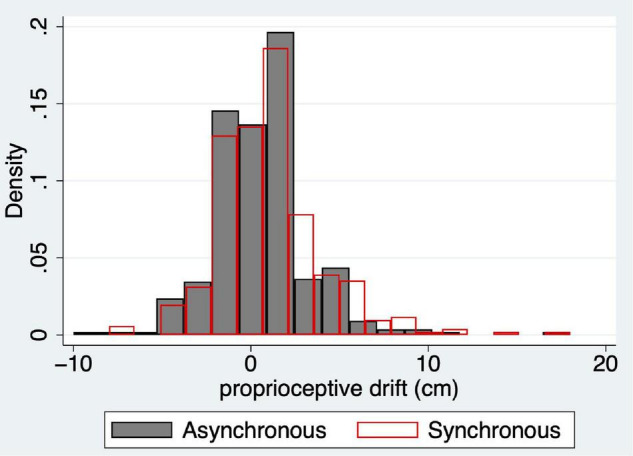
Histograms of proprioceptive drift for the asynchronous (*dpdasync*, grey solid) and synchronous responses (*dpdsync*, red outline) for the full sample (*n* = 353). The probability density is shown on the y-axis of the change in reported right hand location in cm towards the location of the rubber hand (x-axis).

Figure 4 shows the histograms for proprioceptive drift for all 353 cases. At this point, the most important aspect to note is that the distributions appear at first sight to be approximately symmetric around 0, although with an added skew to the right in both cases. We will address this later.

### A Statistical Model for the Questionnaire Data

Here, we present a statistical model that overcomes the three problems mentioned in Section “Normal Linear Regression for the Questionnaire Scores,” namely, (i) the distributions of the response variables do not fit the underlying assumption of normality, (ii) there are influential points, and (iii) the questionnaire response variables are bound to the interval [-3,3], which was not taken into account in the analysis.

Instead of using *ss* and *sa*, these are linearly transformed into the range [0,1] (in fact, [0.01, 0.99] since exact values of 0 or 1 are not tolerated by the model). We call these transformed response variables for *ss* and *sa*, *pss*, *pas* ∈ [0,1], respectively. Instead of the assumption of a normal distribution, we use the Beta distribution as a model for these new bounded response variables, conditional on the parameters. As seen from Figure 2, the observed distributions can take quite different shapes. The Beta distribution was chosen since it can adapt to many different shapes (skewed, J-shaped, reverse-J, U shaped, symmetrical around 0.5 with the mode at 0.5, etc.) depending on the parameters, and it is bound to the [0,1] range.

The Beta distribution has two parameters, α > 0 and β > 0, referred to as the *Beta*(α,β) distribution, which has mean $\frac{\alpha }{\alpha +\beta }.$ The probability density is 0 outside of the range [0,1].

Suppose that *y* is a response variable (*pss* or *pas*) and *x* is a covariate (*swash*). Then, the likelihood (the probability distribution of the data conditional on the parameters) is:


(2) y∼Beta(ϕμ,ϕ(1-μ)),0<y<1

which ensures that the mean is μ. The parameter ϕ > 0 is not of any interest here.

Since μ has to be constrained to be in the interval [0,1], we need a “link function” that relates μ to the linear expression in the covariate, η = β_0_ + β_1_*x* (usually called “the linear predictor”), so that no matter what the value of this expression, μ is in the correct interval. Typical choices are the “inverse logit” function or the cumulative distribution function of the standard normal distribution. We use the first (and the results are almost identical if the second is used). Hence,


(3) $$\mu =\frac{1}{1+e^{-\eta }}$$

The prior distributions chosen are “weakly informative” (Gelman et al., 2008; Lemoine, 2019):


$$\begin{array}{c} \beta_{0},\beta_{1}∼normal\left(mean=0,standarddeviation=10\right)\\ \;\phi ∼Gamma\left(2,0.1\right) \end{array}$$

This means that the prior distributions are proper probability distributions but with wide variance. Note that the prior (equal tail) 95% credible intervals for β_0_, β_1_ are –20 to 20 and for *ϕ* 2.4 to 55.7. Hence, the prior distributions have very wide support, but in any case, because the dataset is large, these priors will be overwhelmed by the data.

For the response variable *pas*, these parameters are denoted β_pas,0_, β_pas,1_, *ϕ*_*pas*_, and for *pss*, β_pss,0_, β_pss,1_, *ϕ*_*pss*_.

We have *n* = 353 observations (*pss*_*i*_, *pas*_*i*_), *i* = 1, 2, *n*. We use the Stan probabilistic programming language (Stan Development Team, 2011–2019; Carpenter et al., 2017)^4^ through the rstan interface (see text footnote 2) to derive posterior distributions for the parameters; i.e., the distributions are updated based on the data. Note that this is one overall model for both *pas* and *pss* simultaneously and not two separate models.

Stan was executed with 2000 iterations using 4 chains. All Rhat = 1, indicating that the 4 chains mixed and converged without problems. The Stan programs that accompany this paper are available and can be executed online–see Supplementary Text 1.

Table 1 summarises the posterior distributions of the parameters. Note that the posterior credible intervals are considerably narrower than the prior credible intervals. Additionally, the posterior distributions of the slopes β_pss,1_ and β_pas,1_ are in the positive region, indicating the positive association between *pas* and *swash* and between *pss* and *swash*. Moreover, from the means of these distributions and the credible intervals, it can be seen that the slopes for *pss* and *pas* are similar. However, considering the intercepts, the probability that β_pss,0_ > 0 is 0.916, whereas the probability that β_pas,0_ < 0 is 1.000. Hence, the results show that the relationship between the response variables and *swash* follows two almost parallel curves, but the asynchronous curve is considerably lower than the synchronous curve at every level of *swash*. This corresponds to the findings of Section “Normal Linear Regression for the Questionnaire Scores.”

**TABLE 1:** Summaries of the posterior distributions of the model showing the means, standard deviations, and 95% credible intervals.

Parameter	Mean	SD	2.5%	97.5%	Prob > 0
**Synchronous (pss)**					
β_*pss*,0_	0.18	0.13	–0.08	0.44	0.916
β_*pss*,1_	0.35	0.08	0.20	0.50	1.000
*ϕ*_*pss*_	2.29	0.16	1.98	2.60	
**Asynchronous (pas)**					
β_*pas*,0_	–0.95	0.14	–1.23	–0.69	0.000
β_*pas*,1_	0.43	0.08	0.28	0.58	1.000
*ϕ*_*pas*_	2.36	0.15	2.08	2.66	

*Prob > 0 contains the posterior probabilities of the parameter being positive.*

**FIGURE 5:**
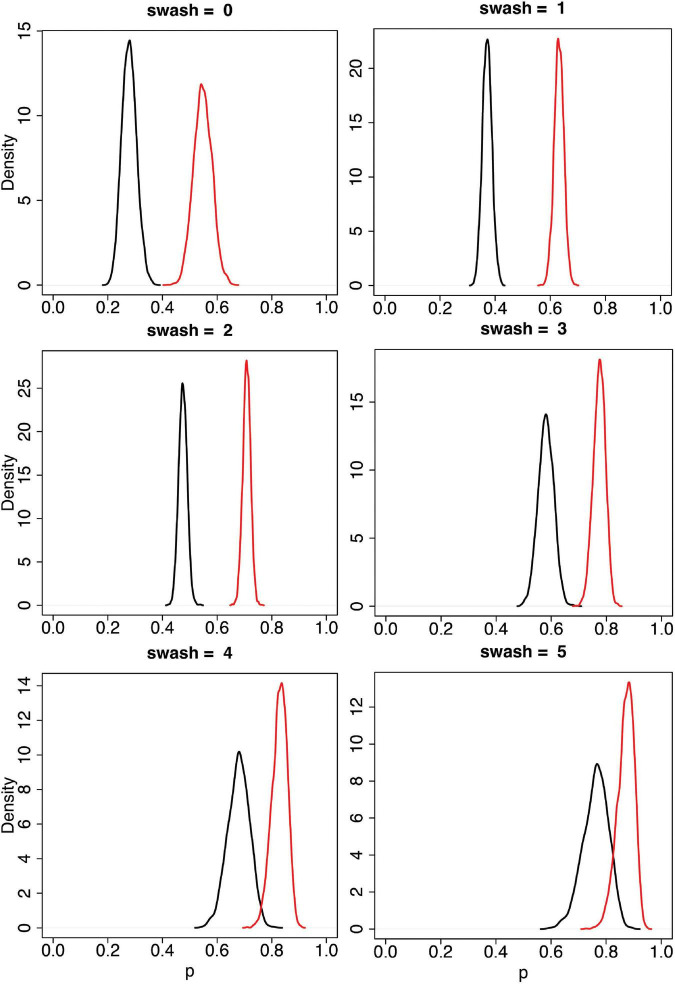
Posterior distributions of the mean for *pss* and *pas* by values of *swash* = 0, 1, .., 5. The black curves are for *pas* and the red curves for *pss*. These are from the Bayesian model (Eqs 2, 3). The horizontal axis (*p*) represents *pss* or *pas*, and the vertical axis is the probability density, so that areas under the curve are probabilities. The posterior distributions are updates from the prior distributions based on the data. As can be seen the posterior distributions are clearly different for the synchronous (red) and the asynchronous conditions (black) for all levels of trait hypnotisability (SWASH) including the extrapolated high SWASH scores 4 and 5.

Using the statistical model (Eqs. 2, 3), we can find the posterior distributions of the mean questionnaire scores (μ) for each of *pas*, *pss* for any level of *swash*, simply as the posterior distribution of μ using η = β_0_ + β_1_ *swash*. Figure 5 shows these posterior distributions for each level of *swash* = 0, 1, …, 5. It is apparent that the asynchronous and synchronous RHI scores increase with increasing values of *swash*, as would be expected from the correlations reported in Lush et al. (2020). However, it is also the case that the distinction between asynchronous and synchronous is maintained at every level of *swash*, with the mean synchronous scores always greater than the mean asynchronous scores with high probability. Table 2 shows the 95% credible intervals for these distributions. The credible intervals do not even overlap except for the highest level of *swash*. Note that in these data, the median level of *swash* is 1.6, and the mean is 1.62. Obtaining a sample of people with very high SWASH scores, such as 4 and 5 (very hypnotisable individuals) in Figure 5, is extremely unlikely to occur by chance when drawn from the general population. Indeed, in the sample of 353 people, the maximum value of *swash* was 3.75, with the 95th percentile 2.88. Nevertheless, our model allows for such extrapolations to be done, and the results indicate that even in such groups with very high SWASH scores a clear effect of multisensory integration is present.

**TABLE 2:** Posterior 95% credible intervals for the mean RHI scores for different levels of *swash*.

*swash*	Asynchronous (pas)		Synchronous (pss)	
0	0.23	0.33	0.48	0.61
1	0.34	0.41	0.60	0.67
2	0.44	0.50	0.68	0.74
3	0.52	0.64	0.73	0.81
4	0.60	0.75	0.77	0.88
5	0.66	0.84	0.80	0.92

The Stan program supports the generation of new simulated data based on the posterior distributions. Pseudorandom observations are drawn from the posterior distributions of the parameters, and Eqs 2, 3 are used to generate new sets of observations on the response variables. This results in what are termed the “predicted posterior distributions” of the response variables. These predicted posterior distributions can be compared with the original data. If the model is adequate, then the predicted posterior distributions should be similar to the observed distributions of *pss* and *pas*.

**FIGURE 6:**
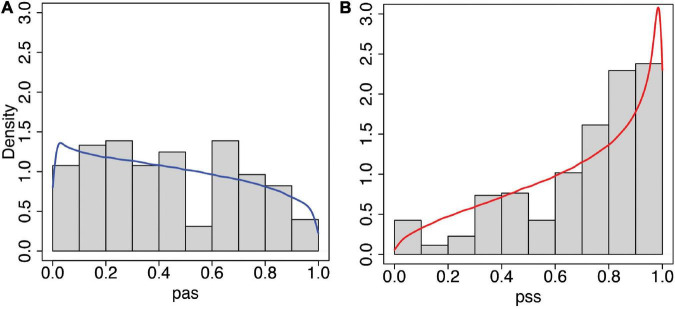
Histograms and predicted posterior distributions of the questionnaire scores from the statistical model (Eqs 2, 3). **(A)** Asynchronous (*pas*) and **(B)** Synchronous (*pss*). In each case, the predicted posterior distributions are the curves shown over the histograms of observed data. The model fits the data well, including the characteristic shift towards higher scores in the synchronous condition. This can be compared with the model that uses a normal distribution shown in Supplementary Figure 1.

The mean ± SD of the observed *pss* is 0.71 ± 0.25, and the corresponding values for the predicted posterior are 0.68 ± 0.26. For *pas*, these values are 0.44 ± 0.27 and 0.44 ± 0.28. Figure 6 shows the histograms of each of *pas* and *pss* and the corresponding predicted posterior distributions. In contrast, Supplementary Text 2 shows equivalent results when a normal distribution is used to model the *sa* and *ss* scores, and the fit is poor (see Supplementary Figures 1A,B).

The overall conclusion is that hypnotisability plays a role since greater values are associated with increases in subjective illusion scores, which is in line with the findings of Lush et al. (2020). However, at every level of hypnotisability, there is a clear distinction between the synchronous and asynchronous scores, with the synchronous scores being substantially greater.

### Statistical Model for Proprioceptive Drift

As mentioned above, Figure 4 shows that the distributions of the proprioceptive drifts seem at first glance to be almost symmetric around 0, but in fact have some right-skewness. This would fit a situation where a component of the response is normally distributed (just a normally distributed random error around 0) but also with an additional effect of the experimental manipulation of synchrony/asynchrony that seems to push the response away from normality towards higher drift scores in the synchronous case. An appropriate distribution to model this situation is the “exponentially modified normal distribution.” This is the distribution of the sum of a normally distributed random variable (say, *x*) and an independent exponentially distributed random variable (say, *y*). Then, *x* + *y* has this distribution. The distribution has three parameters (μ,σ > 0,λ > 0), where μ and σ are the mean and standard deviation of *x*, and λ is the rate of *y* (the mean of *y* is 1/λ). Therefore, setting *z* = *x* + *y*, the mean of the random variable *z* is μ+1/λ.

The proprioceptive drift responses are *dpdsync* and *dpdasync*. If *z* represents either one of those, then the model is:


(4) $$z∼ExpModNormal\left(\mu -\frac{1}{\lambda },\sigma ,\lambda \right)$$

Hence, the mean of *z* is μ. We relate μ to the linear predictor as follows:


$$\mu =\beta_{0}+\beta_{1}swash$$

We use the same non-informative priors for β_0_, β_1_ and σ as above and $\frac{1}{\lambda }∼Gamma\left(2,0.1\right)$ (so that λ has an “inverse gamma distribution”). We denote the mean of the exponential part of the distribution by $\mu_{exp}=\frac{1}{\lambda }$.

The two sets of parameters are β_*pds*,0_, β_*pds*,1_, σ_*pds*_, λ_*pds*_, and μ_*exp*, *pds*_ for synchronous proprioceptive drift (*dpdsync*) and β_*pda*,0_, β_*pda*,1_, σ_*pda*_, λ_*pda*_, and μ_*exp*, *pda*_ for asynchronous drift (*dpdasync*). This model is included as an extension of that presented above for the subjective scores; i.e., there is one overall model that incorporates both the subjective and proprioceptive drift scores. Table 3 shows the summaries of the posterior distributions (this is just an extension of Table 2 since it is from the same overall model).

**TABLE 3:** Summaries of the posterior distributions of the proprioceptive drift model showing the means, standard deviations, and 95% credible intervals.

Parameter	Mean	SD	2.5%	97.5%	Prob > 0
**Synchronous (dpdsync)**					
β_*pds*,0_	0.19	0.35	–0.49	0.82	0.711
β_*pds*,1_	0.54	0.19	0.16	0.92	0.997
σ_*pds*_	2.10	0.14	1.83	2.42	
λ_*pds*_	0.49	0.06	0.40	0.66	
μ_*exp*, *pds*_	2.05	0.23	1.61	2.41	
**Asynchronous (dpdasync)**					
β_*pda*,0_	0.41	0.33	–0.22	1.00	0.895
β_*pda*,1_	0.06	0.18	–0.29	0.44	0.635
σ_*pda*_	2.01	0.12	1.78	2.30	
λ_*pda*_	0.59	0.07	0.47	0.80	
μ_*exp*, *pda*_	1.73	0.20	1.36	2.04	

*Prob > 0 contains the posterior probabilities of the parameter being positive.*

Table 3 shows that in the case of the synchronous stimulation, there is a positive association between the drift and *swash* (the probability of the slope being positive is 0.997, and the 95% credible interval is well into the positive region). In the asynchronous case, there is little evidence of an association between the drift and *swash*, the probability of the slope being positive is only 0.635, and the credible interval well includes 0; in fact, the mean of the distribution is almost 0 with a much larger standard deviation (0.18).

**FIGURE 7:**
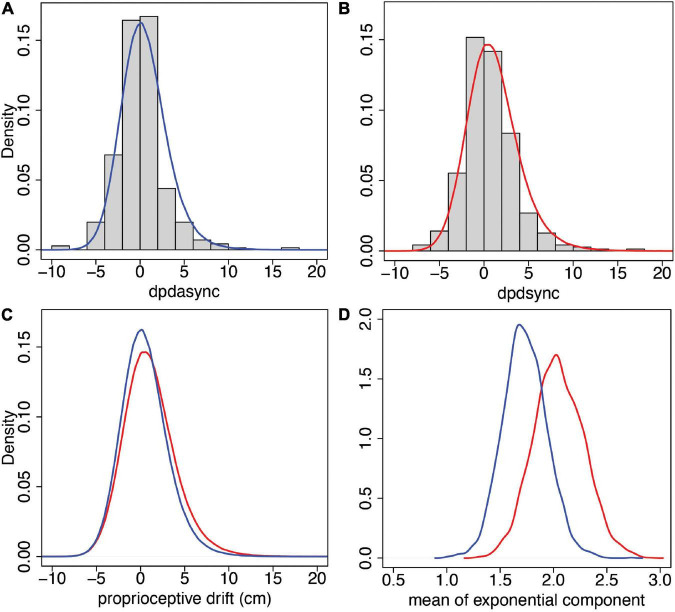
Predictive and posterior distributions for proprioceptive drift from the model of Section “Statistical Model for Proprioceptive Drift” (Eq. 4). **(A)** The histogram of the observations and predictive posterior for *dpdasync*. **(B)** The histogram of the observations and predictive posterior for *dpdsync*. **(C)** The predicted posteriors of *dpdasync* (blue) and *dpdsync* (red). **(D)** The posterior distributions of the means of the exponential components for asynchronous (blue) and synchronous (red) conditions.

The predictive posterior distribution for synchronous proprioceptive drift (*dpdsync*) has mean ±SD of 1.06 ±2.98, and the corresponding observed values are 1.05 ±3.02. For the asynchronous case (*dpdasync*), the predicted posterior distribution values are 0.51 ±2.67, and the corresponding observed values are 0.49 ± 2.73. Figures 7A,B show the predicted posterior distributions superimposed over the corresponding histograms of the observed data. Figure 7C shows the comparison of the predicted posterior distributions for the two conditions, and it can be seen that the synchronous and asynchronous distributions are very similar. However, Figure 7D shows the posterior distributions of the means of the exponential components only, μ_*exp*, *pds*_ and μ_*exp*, *pda*_ showing greater probabilities for higher values for the synchronous case. For example, the posterior probability,


$$P\left(\mu_{exp,pds}>1.75|data\right)=0.898,whereas$$

$$P\left(\mu_{exp,pda}>1.75|data\right)=0.455,and$$

$$P\left(\mu_{exp,pds}>\mu_{exp,pda}|data\right)=0.850.$$

Overall, the exponentially modified normal distribution fits these proprioceptive drift observations well. Moreover, the model shows that the exponential part, which accounts for departures from symmetry around 0, has a greater probability of higher mean values for the synchronous stimulation than for the asynchronous stimulation. This is a further demonstration of the impact of the different types of multisensory stimulation, with the evidence favouring greater proprioceptive drift for the synchronous condition than for the asynchronous condition.

### Model Checking

The final point to consider for the overall model that incorporates both the questionnaire and proprioceptive drift responses is to assess its predictive capability and whether it might include influential points. We use the “leave-one-out” (loo) method (Vehtari et al., 2017), which leaves out each data point in turn, fits the model with the remaining data, and estimates the one left out. This provides an “out-of-sample” estimate of fit (i.e., each “left-out” data point is not used to estimate the model that then predicts it). This results in a statistic (ELPD, expected log pointwise predictive density) that showed no problems with the convergence of the model. It also finds “Pareto k estimates” corresponding to each data point, where a large value indicates a potentially outlying or influential point with respect to the model (i.e., not well predicted by the remaining data). The requirement for a good fit is that values of *k* < 0.5 and *k* < 0.7 is acceptable. The method also estimates a statistic that indicates whether there was overfitting of the data through an estimate of the number of parameters.

For each response variable and all data points except one, the Pareto k estimates are less than 0.5, with one between 0.5 and 0.7 for *dpdasync*. Unlike the case of the original normal distribution regression where there were a large number of influential points, in this analysis, there were none. Moreover, no overfitting is indicated, and all estimates of the numbers of parameters are close to the actual number. Overall, the model has good predictive capability.

## Results of an Alternative Model

### Comparing the Multisensory Condition With Swash

In the above, we followed the analysis conducted by Lush et al. (2020), who treated the synchronous condition questionnaire scores separately from the asynchronous scores, albeit we have done this within one overall model. However, their experiment followed a within-group design in which each participant experienced asynchronous or synchronous conditions in counterbalanced order. The advantage of analysing these data within groups is that we can directly compare the differing effects of the multisensory condition (asynchronous, synchronous) with *swash* in their impact on the questionnaire results. This gives rise to a simple mixed effects model. We use the following notation, for *i* = 1, 2, …, *N* (= 2×*n*), *n* = 353, the number of participants, where:

*ps*_*i*_ is the normalised questionnaire score in the [0.01, 0.99] range as above.

*cond*_*i*_ = 0 for the asynchronous condition and 1 for the synchronous condition.

*swash*_*i*_ is the corresponding *swash* score.

*id*_*i*_ is the identifier number for the corresponding participant, where *id*_*i*_ is in the range 1, 2, …, *n* for the *n* participants.

Then, the linear predictor is:


(5) $$\begin{array}{c} \eta_{i}=u_{id_{i}}+\beta_{0}+\beta_{1}cond_{i}+\beta_{2}swash_{i}+\beta_{3}\left(cond_{i}\cdot swash_{i}\right)\\ i=1,2,\ldots ,N \end{array}$$

The inverse link function is given by Eq. 3


(5) $$\mu_{i}=\frac{1}{1+e^{-\eta_{i}}}$$

and the likelihood is, following Eq. 2:


(6) $$ps_{i}∼Beta\left(\phi \mu_{i},\phi \left(1-\mu_{i}\right)\right)$$

where μ_*i*_ is the mean.

The term *u*_*id_i_*_ in Eq. 5 expresses the random effects part of the model, which takes into account the fact that there are a pair of observations for each participant and *u*_*id_i_*_ will be the same for that pair, also allowing for individual differences. The remaining part of Eq. 5 is for the fixed effects and allows for the questionnaire scores to be influenced by condition, *swash*, and the interaction between them.

The prior distributions of β_*j*_ are *normal*(*mean* = 0, *standard deviation* = 10), giving prior 95% credible intervals in the range ± 20. The prior distribution for ϕ is *Gamma*(2,0.1) and therefore the prior 95% credible interval is 2.4 to 55.7, as used earlier. Convergence of the model was achieved with prior distributions for $u_{id_{i}}∼normal\left(0,\frac{1}{4}\right)$ hence with prior 95% credible intervals ± 0.5.

The model was fit with Stan using 4000 iterations and 4 chains, and convergence was achieved with all Rhat = 1, indicating that the chains had properly mixed. More iterations were used here because of the greater number of parameters. The summaries of the posterior distributions of the parameters are shown in Table 4.

**TABLE 4:** Summaries of the posterior distributions of the model (Eqs. 5, 6) showing the means, standard deviations, and 95% credible intervals.

Parameter	Coefficient of	Mean	SD	2.5%	97.5%	Prob > 0
β_0_		–0.96	0.13	–1.23	–0.69	
β_1_	*cond*	1.13	0.19	0.77	1.49	1.000
β_2_	*swash*	0.43	0.08	0.28	0.58	1.000
β_3_	*cond*⋅*swash*	–0.06	0.10	–0.26	0.14	0.278
ϕ		2.52	0.13	2.28	2.77	

*Prob > 0 contains the posterior probabilities of the parameter being positive.*

Table 4 shows the clear effect of both the multisensory condition and *swash*. There is no useful contribution of the interaction term, which will not be considered further. The size of the coefficients (the “Mean” column) shows that condition has a greater impact than *swash* (the coefficient is 2.63 times larger for *cond*), and the 95% credible intervals do not overlap. However, the different impacts of *cond* and *swash* can be more clearly seen by considering the posterior predicted distribution of *ps*.

**FIGURE 8:**
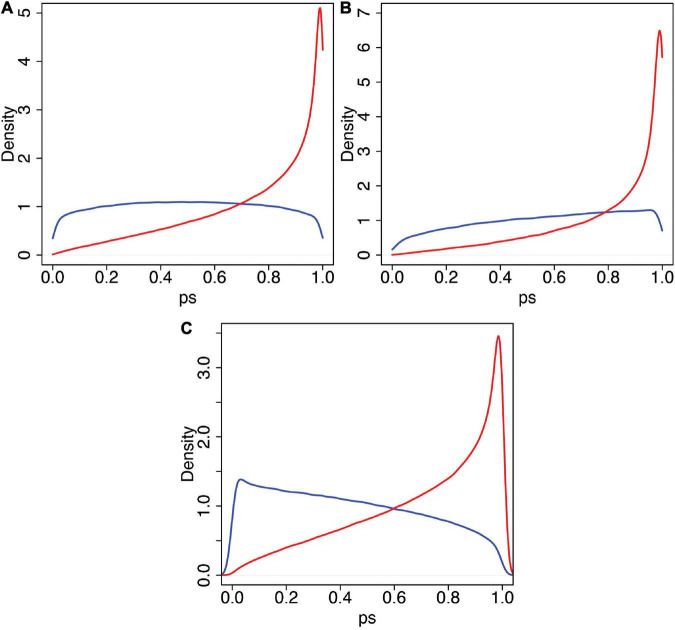
The predicted posterior distribution of *ps*. **(A)** For values of *swash* > 1.60 (the median). **(B)** For values of *swash* > 2.64 (the 90th percentile). **(C)** For *expect* > 1. In each case, the blue curve is for asynchronous, and the red curve is for synchronous. Note that the characteristic difference between conditions is evident in each case, with the synchronous condition always being associated with higher probability (density) of high RHI scores across the two levels of trait hypnotisability (*swash*) displayed.

Recall that the 50 and 90% quantiles for *swash* are 1.6 and 2.64, respectively. Figure 8 shows the posterior predicted distributions of *ps* for *swash* > 1.6 (Figure 8A) and for *swash* > 2.64 (Figure 8B). In each case, the distributions for asynchronous and synchronous stimulation are shown. It is very clear that the probability density is concentrated towards greater values of *ps* in the synchronous case compared to the asynchronous case, irrespective of *swash*.

A high questionnaire score of 2 on the –3 to 3 scale corresponds to *ps* > 0.827. Table 5 shows the posterior probabilities that *ps* > 0.827 for combinations of *swash* greater than its median and 90th percentile and the asynchronous and synchronous conditions. Once again, it is clear that the dominant factor is the condition, which increases by a factor of 3.25 going from asynchronous to synchronous in the median *swash* case and by 2.64 in the 90th percentile case. In contrast, the probability increases by a factor of 1.47 going from median to 90th percentile *swash* in the asynchronous case and 1.2 in the synchronous case. The evidence is strong that the multisensory factor contributes to a greater extent than *swash*.

**TABLE 5:** *P*(*ps* > 0.827|*swash* > *s*, *cond* = *c*) for *s* = 1.6, 2.64 and *c* = 0,1.

	swash > 1.6 (median)	swash > 2.64 (90th %tile)
*cond* = 0 (asynchronous)	0.153	0.225
*cond* = 1 (synchronous)	0.497	0.594

### The Impact of Expectancy

Lush et al. (2020) also reported a correlation between expectancy illusion ratings and actual illusion ratings in the synchronous condition (*R*^2^ = 0.06: *p* < 0.001), and based on this, together with another paper (Lush, 2020), that we discuss in Supplementary Text 4, concluded that expectations contribute to the RHI and that they play an important role by triggering “phenomenological control” and “hypnotic hallucinations” to meet those expectancies. However, they did not pay much attention to the asynchronous condition or examine whether differences in expectancies between the synchronous and asynchronous conditions could explain the condition-specific differences in RHI ratings, nor assess the relative contribution of the expectancy effect. However, it is possible to consider this within our model by taking into account the impact of the expectancy scores following the manipulation. Participants were asked “How strongly do you expect to feel the rubber hand is your own hand at least a little bit when the brush strokes on your own hand and on the rubber hand are in synchrony?” (*expectancy_sync*) and the same question for asynchronous with the only difference that words “are in synchrony” had been replaced with “are not in synchrony” (*expectancy_async*). These were scored as –3 (strongly disagree) to 3 (certain). We consider the difference, *expect* = *expectancy*_*sync-expectancy*_*async*, as an overall measure of expectation in favour of experiencing the RHI in the synchronous as opposed to the asynchronous condition (Note that there are two missing values in the expectancy scores, and these were replaced by 0 for *expect*).

The model in Eq. 5 can be extended to include the new variable:


(7) $$\begin{array}{c} \eta_{i}=u_{id_{i}}+\beta_{0}+\beta_{1}cond_{i}+\beta_{2}swash_{i}+\beta_{3}expect_{i}\\ \;+\beta_{4}\left(cond_{i}\cdot expect_{i}\right) \end{array}$$

Here, we have included a main effect for *expect* and its interaction with *cond*. Checks of a model that included interactions of *swash* with *cond* and *expect* with *swash* show that these do not contribute to the model fit and can be excluded. No interaction between *swash* and *expectation* is itself noteworthy since it does not seem to fit with Lush and colleagues’ assumption that expectations and trait hypnotisability interact.

Table 6 shows the results for this model. For *cond* and *swash*, the results are qualitatively the same as in Table 4, as would be expected if multisensory condition and trait suggestibility are the most important factors. There is little evidence of a main effect for *expect* (the main effect corresponds to the asynchronous condition), but the interaction with *cond* is important. What this shows is that in the synchronous condition only, the greater the value of *expect*, the greater the illusion. This is not surprising and shows an overall consistency of the model: the more that participants believed that they would experience the RHI based on the instructions they received before the experiment, the more likely they would be to report this. However, this does not mean that “demand characteristics” are the cause of the illusion or that expectations play a significant role. Note that there was no effect of expectation in the asynchronous condition, suggesting that elimination of the RHI by the temporally incongruent visual and tactile signals was so decisive that it overrode any prior expectations regarding the illusion.

**TABLE 6:** Summaries of the posterior distributions of the model with the linear predictor (Eq. 7) showing the means, standard deviations, and 95% credible intervals.

Parameter	Coefficient of	Mean	SD	2.5%	97.5%	Prob > 0
β_0_		–0.89	0.12	–1.12	–0.67	0.000
β_1_	*cond*	0.92	0.10	0.73	1.12	1.000
β_2_	*swash*	0.40	0.06	0.30	0.51	1.000
β_3_	*expect*	–0.02	0.04	–0.11	0.06	0.299
β_4_	*cond*⋅*expect*	0.11	0.06	–0.01	0.23	0.968
ϕ		2.54	0.13	2.30	2.79	

*Prob > 0 contains the posterior probabilities of the parameter being positive.*

The variable *expect* in principle ranges between –6 (when *expectancy*_*sync*_ = −3 and expectancy=async3) and 6 (when *expectancy*_*sync*_ = 3 and *expectancy*_*async*_ = −3). There are only 39 cases with *expect* > 2 but 128 cases with *expect* > 1. Figure 8C shows the predicted posterior distributions of *ps* for *expect* > 1 under asynchronous and synchronous conditions. Again, the evidence is strong that it is the multisensory condition that dominates the results; the probability of higher scores is much greater in the synchronous than in asynchronous conditions, even at this relatively high level of *expect*. The same shaped distributions are obtained even for greater levels with *expect* > 2, *expect* > 3, *andexpect* > 4 (which is the highest that we can include in the model based on the observed data; results not shown).

### Comparing Models

Although we have seen that both hypnotisability and expectations may contribute to the subjective RHI scores, is it worth including these in the statistical model, i.e., how much do they contribute in comparison to the multisensory factor? To address this issue, we use the ELPD statistic introduced earlier. ELPD is a pointwise predictive log density for a new dataset (Vehtari et al., 2017). It is a cross validation method that is based on carrying out a model fit with all data points but one and then seeing how well the one left out can be predicted. This is carried out for each data point in turn. The resulting ELPD is a combined log probability that estimates the predictive power of the model. It is especially important because its estimates are always based on “external” data in the sense that the one left out is not part of the dataset that is used to predict it. The higher the value of the ELPD the better the predictive power of the model.

Table 7 shows the ELPDs and their differences across several different models. A simplified notation is used for each model. For example, Model 1 corresponds to Eq. 7. Interaction terms that have no effect are not included. It can be seen that Model 1 has the greatest ELPD. However, dropping the terms involving *expect* (Model 2) leads to hardly any change in the ELPD, the change being –0.5 with a comparatively large standard error of 2.0. Therefore, the more parsimonious Model 2 is preferred to the more complex Model 1. If we drop *swash* from Model 2 to obtain Model 3, then there is a noticeable drop in the ELPD (-24.7 with a standard error of 7.3 that is approximately 3 times smaller), indicating that Model 2 is preferred. However, if we drop *cond* from Model 2 to arrive at Model 4, then there is a much larger decrease in the ELPD (-81 with a standard error of 12.3 that is almost 7 times smaller). This discussion emphasises what we have found earlier: both *cond* and *swash* contribute to the illusion scores, but *cond* is notably the most important factor, and *expect* only makes a negligible contribution. Models 5 and 6 are included for completeness and show that without the inclusion of *cond*, the ELPD is very much smaller.

**TABLE 7:** Comparison of ELPD across several models.

Model	ELPD	S.E. ELPD	ELPD difference	S.E. difference
1. *cond* + *swash* + *expect* + *expect*×*cond*	126.5	14.9	0.0	0.0
2. *cond* + *swash*	125.9	15.0	–0.5	2.0
3. *cond*	101.8	13.6	–24.7	7.3
4. *swash*	45.5	8.9	–81.0	12.3
5. *swash* + *expect*	45.2	9.0	–81.2	12.4
6. *expect*	25.6	6.5	–100.9	13.7

*The columns are the ELPD, its standard error, the pairwise difference, and the standard error from the model with the max ELPD.*

Overall, as shown in this section, it is clear that the dominant factor in the illusion scores is the multisensory one. Hypnotisability contributes to some extent, while expectations contribute negligibly.

## Discussion

A series of recent papers has challenged the traditional explanation of the RHI being a perceptual bodily illusion based on multisensory integration and instead proposed an explanation mainly around hypnotic suggestibility, expectations, and demand characteristics, all of which are primarily based on the same underlying data that we have discussed (Lush et al., 2020; Seth et al., 2021; Lush and Seth, 2022). For example, Lush et al. (2020) claimed that the RHI “may or may not be entirely attributable to demand characteristics and phenomenological control” (p5), that “demand characteristics can drive experience and that these effects are driven by the control of phenomenology to meet task expectancies according to a stable trait ability” (p2). In our extensive reanalyses of the same data the major conclusion is that although hypnotisability plays some role in the intensity of reported subjective RHI, the effect of synchronous and asynchronous stimulation is dominant and independent of the suggestibility trait, therefore supporting the multisensory explanation. Moreover, our analyses reveal that expectancies make a negligible contribution compared to the impact of multisensory integration (and suggestibility). We also find little evidence that hypnotisability influences proprioceptive drift. Collectively, the current findings are not in line with Lush and colleagues’ strong claims regarding expectations and trait hypnotisability but fit better with the established view that the RHI is a perceptual bodily illusion driven primarily by mechanisms related to multisensory integration.

Using the same normal-based regression analysis carried out by Lush et al. (2020), we find that when the intercepts of the regression are considered as well as the slopes, synchronous subjective RHI scores are predicted to be greater than asynchronous at every level of hypnotisability (*swash*). Second, eliminating the linear effect of *swash* from the subjective response variables shows a substantial difference in the distributions of the synchronous and asynchronous scores (Figure 1). Third, with respect to proprioceptive drift, the regression lines show that at every level of *swash*, the synchronous drift is predicted to be higher than the asynchronous drift [“predicted” is the terminology used by Lush et al. (2020)].

Next, only examining histograms of the raw data for different levels of *swash* (Figure 2) shows important differences between the distributions of the subjective illusion scores between the synchronous and asynchronous conditions. Moreover, for every level of *swash*, the probability of a high illusion score is greater for synchronous than asynchronous (Figure 3).

However, there are problems in using a normal distribution: none of the response variables or residual errors after regressions follow a normal distribution, and using this model involves multiple influential and outlying points (Figures 2C,D). Therefore, we carried out an alternative (Bayesian) analysis where the predicted posterior distributions generated from the derived model substantially fit these observed data (Figures 6, 7). We confirm that at every level of *swash*, illusion scores are likely to be greater for the synchronous than the asynchronous condition (Figure 5). We also find that proprioceptive drift can be analysed as the sum of a normal and exponential distribution, and the mean of the exponential component is greater for the synchronous than the asynchronous condition (Figure 7D).

Finally, by analysing the impacts of *swash* and expectancy ratings compared to the contribution of multisensory conditions and by systematically comparing models that include or do not include the variables condition, *swash* and expectancy, we find that the influence of multisensory condition dominates. The multisensory condition makes the strongest contribution by far to the subjective RHI score, i.e., two to three times more than that of other factors; hypnotisability has some effect, but the contribution of expectation is so small that it can be ignored. Clearly, these results do not support the view of Lush and colleagues that hypnotisability and expectations may play the most important role in the RHI.

The overall conclusion from our analysis is that although the correlations reported by Lush et al. (2020) are, of course, correct, they provide an incomplete account of the relationship between the SWASH scale and the RHI questionnaire results and proprioceptive drift. Not taking into account the intercepts of the regressions, nor the fact that at increasing levels of *swash* the difference between synchronous and asynchronous is maintained, nor the difference in distributions of the questionnaire scores after eliminating the linear effect of *swash*, gives the impression that *swash* is a causal factor (perhaps the only one) in explaining the RHI, even though, of course, we know that correlation does not imply cause. Our analysis shows that this approach ignores the major contribution of multisensory integration. Since multisensory integration was the only factor that was manipulated in the experiment (barring the expectation induction that had no effect), this must be a causal factor. Causality, in this case, is further supported by the facts that the specific pattern of multisensory information that triggers the illusion *precedes* the onset of the subjective illusion and that there is a strong hypothesis regarding the underlying *mechanism*, both at the computational and neural implementation levels (Graziano, 1999; Graziano et al., 2000; Kilteni et al., 2015; Samad et al., 2015; Fang et al., 2019; Guterstam et al., 2019; Chancel et al., 2021a).

Altogether, therefore, the results from the current new analyses fit well with the view that the rubber hand illusion is a multisensory perceptual bodily illusion. The difference in illusion ratings between the synchronous and asynchronous conditions is present for every level of hypnotisability (as measured by the SWASH scale). That is, this difference is present in both the least and the most hypnotisable individuals in the sample, which is interesting, as it suggests that hypnotisability is not a necessary factor for experiencing the RHI and that even in the most hypnotisable individuals studied, there is a major effect of multisensory integration. Notably, even though the level of illusion is affected by SWASH, the level of illusion is influenced by the multisensory condition independent of SWASH, and as our model comparisons show, the contribution of multisensory integration is always greater than the hypnotisability factor, even at the highest levels of the SWASH scale.

The fact that the relationship in illusion ratings between the synchronous and asynchronous conditions is stable across SWASH is interesting in several ways, beyond the points that have already been discussed. First, this stability suggests that the relationship between expectations/demand characteristics and hypnotic suggestibility may not be as tight as Lush and colleagues theorised. If the idea is that the participants produce experiences and behaviour to meet task expectancies, then, if you are not suggestible, you should not respond very differently to the synchronous and asynchronous conditions. However, if you are highly suggestible, you should respond very differently because you have a personality that will make you respond to task demands with the ability to generate “experiences” and the behaviour to do so convincingly. However, as the results from the current analyses show, there is no evidence for such an interaction between the multisensory condition and trait hypnotic suggestibility, which is also in line with Lush et al. (2020) and Ehrsson et al. (2022). Second, the fact that the difference in illusion measures between conditions is not related to suggestibility has a bearing on the interpretation of many previous findings in the RHI literature and for the design of future studies. We now know that previous RHI studies that used a within-subject design and reported condition-specific differences in RHI measures between synchronous and asynchronous conditions (and they are many) have reported findings that are probably not confounded by trait hypnotic suggestibility. Similarly, for future RHI studies that seek to control and eliminate the effect of hypnotic suggestibility, the results underscore the effectiveness of a control condition.

Furthermore, for between-group comparisons, the current results suggest that it may be important to try to match the level of suggestibility in the different groups if there are reasons to assume that they may differ in this dimension (e.g., when comparing certain clinical groups to healthy controls) or to include a common control condition in the different groups and analyse group ×condition interactions. However, randomisation in the selection of groups would result in a very low probability that two groups might differ on their mean level of SWASH if drawn from the same population. Based on the current data, it is extremely unlikely that two randomly selected groups for a between-group study (drawn from a population with similar characteristics as the sample of 353) would have differences in SWASH that are so large that it would bias the results. For example, the chance that two groups of 30 participants would differ in their mean SWASH score by at least 1 is less than 2.7×10^−7^, while the chance of the groups differing by at least 0.5 has a probability of 0.010 (see Supplementary Text 3).

In Lush et al. (2020), the importance of the negative finding regarding SWASH and the condition-specific illusion effects were very much toned down. The authors’ main justification for this was that the importance of the synchronous versus asynchronous comparison was supposedly not motivated by the literature. They claimed that “asynchronous condition measures are typically used only in a prior check that suggestion and compliance effects have been controlled” (p4, Lush et al., 2020). However, control conditions, such as the asynchronous condition, have been an integral part of the experimental design of RHI studies over the last 20 years, similar to how controls are critical in any area of science; the importance of control conditions is clearly emphasised in reviews of the RHI and similar illusions (Makin et al., 2008; Tsakiris et al., 2010; Blanke et al., 2015; Kilteni et al., 2015; Riemer et al., 2019; Ehrsson, 2020). To be clear, for us, there is no problem in the fact that Lush and colleagues also analysed the conditions in isolation or that they performed *post hoc* exploratory analyses. However, in our view, the comparison of the illusion and control conditions was not sufficiently discussed and apparently not weighted in when formulating the overall conclusion.

All the previous findings and arguments notwithstanding, Lush and colleagues maintain that convincing evidence favouring the RHI as a multisensory illusion is lacking and that demand characteristics provide a more probable and straightforward explanation for the synchronous versus asynchronous differences (Lush et al., 2020; Seth et al., 2021; Lush and Seth, 2022). Demand characteristics refer to the “artefact” that participants can sometimes change their behaviour to meet the expectations of the study and the researchers’ hypothesis (Orne, 1962). In other words, participants may simply be lying, faking, actively imagining or “role playing” to please the experimenter and to be “good subjects” acting in line with the hypothesis. However, in the review by Weber and Cook (1972) of demand characteristics, the authors argued that evidence for demand characteristics in experimental psychological studies is weak and ambiguous in most cases and that convincing evidence for instances where being a “good subject” explains the results is lacking. Furthermore, these authors suggested that participants typically want to follow instructions well rather than to support a particular experimental hypothesis. They argued that a good way to test whether participants in an experiment are “good subjects” is to tell them about the hypothesis in advance and see if that changes the results. Lush and colleagues tested this manipulation by dividing the current sample into three subgroups; approximately one-third of the participants were informed that the synchronous condition would elicit the RHI, the second third were informed that the asynchronous condition would do so, and the third group was given no information about the hypothesis. However, no reliable differences in illusion questionnaire ratings (nor proprioceptive drift) were observed between the groups (Lush et al., 2020; Iriye and Ehrsson, 2022). This outcome suggests that neither demand characteristics nor the “good subject” effect were an important issue, which is in line with the view that such effects are rare and that most participants in RHI experiments will try to truthfully report their experiences as well as they can.

Although it is difficult to completely rule out the theoretical possibility of demand characteristics in any experimental psychological study (e.g., a small one), the key issue is whether such effects can explain the main findings when control conditions and other aspects of the experimental design and task instructions are taken into account. In our view, it is extremely unlikely (most likely impossible) that demand characteristics can constitute the main explanation for the large and replicable RHI effects found across a wide range of paradigms, procedures, and measures in the previous literature. The total number of studies, types of experimental designs and control conditions, and specific findings are simply too numerous to be explained away by demand characteristics, and the literature is too vast to review here; an interested reader is directed to the many previous review articles on this topic (Makin et al., 2008; Tsakiris, 2010; Ehrsson, 2012; Blanke et al., 2015; Kilteni et al., 2015; Riemer et al., 2019; Ehrsson, 2020).

That said, let us briefly consider a few examples of where it is particularly difficult for the participants to determine the underlying hypothesis (see Supplementary Text 4 for more detailed information). In behavioural studies that have used subtle small stepwise manipulations of the degree of asynchrony (or other multisensory incongruences, such as spatial incongruence), it is difficult for participants to know at what level of multisensory incongruence the illusion should start to break down and under which levels it should not change much (Lloyd, 2007; Shimada et al., 2009; Tsakiris et al., 2010; Ide, 2013; Chancel and Ehrsson, 2020). Similarly, the effect of visual noise on the RHI detection task described by Chancel et al. (2021a) is unintuitive and very hard to guess; the addition of noise leads to an observed widening of the visuotactile delays that elicit the illusion that follows a particular function over increasing delays according to a Bayesian causal inference model of multisensory integration. Other noteworthy examples are the hypothesised effects on the cross-modal congruence task across various experimental conditions (see Section “Introduction”) and the effects on force attenuation as quantified with psychophysics in self-touch paradigms (Kilteni and Ehrsson, 2017); how the perception of force should change across the various RHI conditions used in Konstantina Kilteni’s experiments probably cannot be determined by participants. Finally, in neuroimaging studies, the participants do not know which specific multisensory areas should be activated, and even if they did know, people cannot selectively control their level of brain activity in specific areas of the association cortex. For further examples and discussion, see Supplementary Text 4, which also includes an in-depth discussion of the limitations of Lush (2020), which is a study that was offered as a key argument in favour of the authors’ view that demand characteristics are a major explanatory factor for the RHI.

Nevertheless, in our reanalysis of the Lush et al. (2020) data and in the original article, relationships between the SWASH scale and illusion ratings are observed in both conditions. Therefore, how should we interpret this result, and how can hypnotisability influence RHI reports? One possibility could be that hypnotisability modulates the illusion in both conditions to a similar degree and across all levels of SWASH (as suggested by Figure 5). This modulation could in principle occur at perceptual levels of processing, at the metacognition level, or at more general cognitive processing levels. At the perceptual level, hypnotisability may influence the perceptual illusion experienced by influencing bottom-up processing of sensory signals (e.g., signal strength, reliability) or top-down factors (e.g., prior knowledge, attention) that can modulate the multisensory integration processes. One speculative possibility could be that hypnotisability boosts the visuoproprioceptive integration of visual information from the rubber hand and the proprioceptive information from the hidden real hand, which could in theory lead to the augmentation of the illusion in both conditions, which is in line with the current results. One way of testing this possibility in future studies would be to include an additional control condition that eliminates the visuoproprioceptive integration (for example, by rotating the rubber hand 180 or 270 degrees counterclockwise) and checking if the relationship with SWASH is also observed for this control condition.

Alternatively, trait hypnotisability could act postperceptually on metacognition. This could, for example, be accomplished through affecting the internal decision criterion that participants use when they fill out the questionnaire ratings scales and have to judge whether or not they felt the illusion and how certain they are about this judgement (how strongly they agree or disagree with the statements). It is possible that individuals with low scores on SWASH might be more conservative, i.e., that they require a stronger illusion before they will affirm it, and at the same time be more confident when they reject the illusion in the asynchronous condition (being more likely to give very low ratings). Conversely, participants with high SWASH scores may be more liberal when rating the illusion and more willing to give higher scores. Links between trait hypnotisability and alterations in metacognition have been reported in agency tasks (Terhune and Hedman, 2017), thereby providing some indirect support for this idea. It is also possible that highly hypnotisable individuals might more often spontaneously engage in mental imagery and simply imagine that the rubber hand is theirs (in both conditions). When asked to fill out the questionnaires after the RHI, one can speculate that the memories of these acts of imagination might bias the ratings of the true memories of the perceptual illusion experience.

Finally, it cannot be excluded that the relationship between SWASH and questionnaire ratings across conditions may be unrelated to the RHI, both perceptually and at the level of metacognition of bodily awareness. Instead, the relationship might reflect unspecific cognitive biases that in principle could apply to any statements about conceivable or inconceivable unusual experiences related or unrelated to the RHI. One observation that is in line with this view is the significant correlations between SWASH and the control statement in the questionnaire: “The rubber hand began to resemble my own (real) hand, in terms of shape, skin tone, freckles or some other visual feature” (S4), which is observed both in the synchronous condition (*P* = 5.2×10^−10^, *R*^2^ = 0.10, *n*×352 due to 1 missing value) and in the asynchronous condition (*P* = 2.2×10^−11^, *R*^2^ = 0.12) (Lush et al., 2020). Notably, if the S4 ratings are subtracted from the illusion ratings in the synchronous condition, then no relationship to SWASH is observed (Ehrsson et al., 2022). This indicates that SWASH might affect the control statement S4 and the illusion statements in a similar way. Importantly, the RHI is not a visual perceptual illusion, so statement S4 should mainly reflect response bias, suggestion, confabulation, or visual imagery rather than the changes in perceptual bodily awareness that are the hallmark of the RHI. However, only a single control statement was included in Lush et al. (2020), so we do not know how SWASH would correlate with other kinds of control statements. Nonetheless, the observation that SWASH correlates with all statements in all conditions in Lush et al. (2020) means that the alternative hypothesis of non-specific effects of trait hypnotisability on RHI questionnaire statements cannot be ruled out (Ehrsson et al., 2022). Future work is needed to examine this issue in more detail; in such studies, it would be good to include genuinely naïve participants and test the RHI and SWASH on different days (ideally RHI first) instead of conducting hypnotisability screening and the expectancy test procedures directly before the RHI, which may create cognitive bias and spurious correlations due to order effects.

However, Lush et al.’s interpretation is that S4 reflects “visual hallucinations” (Lush et al., 2020) and that trait hypnotisability can explain both “bodily hallucinations” (the RHI) and “visual hallucinations” (Lush and Seth, 2022). Unfortunately, Lush et al.’s dataset included no additional control statements as mentioned above, so how can we know whether the correlations reflect “hallucinations” as opposed to any other factor or cognitive bias?

This brings us to perhaps the most fascinating idea in Lush and colleagues’ “phenomenological control” theory, namely, that many healthy individuals can hallucinate, i.e., see, hear, feel and sense things that are not there, as vividly as real perception. The idea that people can “control their own phenomenology” in this way is inspired by the hypnosis literature and findings from experiments on highly hypnotisable individuals who are actively induced into a hypnotic state or exposed to hypnotic suggestions (Kosslyn et al., 2000; Raz et al., 2002; Oakley and Halligan, 2013). However, exactly what people experience while under hypnosis and how similar or different those experiences are from veridical perception and mental imagery is still not fully understood and an active area of research. The problem with respect to the current debate, as we see it, is that Lush and colleagues extrapolated the concept of “hypnotic hallucinations” from work on highly hypnotisable individuals to the case of typical participants undertaking RHI experiments without critically discussing the underlying assumptions and limitations. One potentially critical issue is that the participants in Lush et al. (2020) were a group that scored low on the hypnotisability trait. As noted above, the mean SWASH hypnotisability score was only 1.6 (*SD* = 0.7) on a scale from 0 to 5 (Lush et al., 2020), which means that most participants in this study were not very hypnotisable and thus were unlikely to be able to experience vivid perception-like “hallucinations.” It has been suggested that the ability to experience “true” hypnotic hallucinations is a rare trait that is only seen in a few individuals who score very highly on hypnotisability scales, similar to synaesthesia (Kallio, 2021). In addition, hypnotisability scales such as SWASH have been criticised for being confounded by purposeful imagination (rather than automatic experiences and behaviours), and it has been argued that there is no single suggestibility trait but rather a set of complementary skills and traits involved (Kallio, 2021). Thus, differences in SWASH scores might relate to cognitive factors other than the ability to produce “genuine” hypnotic hallucinations. In our view, the possibility that a significant number of psychology undergraduates taking part in Lush’s RHI experiments were hallucinating is highly improbable.

In conclusion, none of the current findings of our reanalysis rule out the effect of trait hypnotisability on RHI reports, as has been shown before (Haans et al., 2012; Marotta et al., 2016). However, there is no incompatibility between the argument that hypnotisability influences the strength of the subjective RHI and the view that the RHI is a genuine multisensory bodily illusion because, at every level of hypnotisability, the subjective reports of the illusion and the associated proprioceptive drift are stronger in the synchronous condition than in the asynchronous one, and the multisensory condition clearly dominates in the model fit compared to both hypnotisability and expectations. Thus, we conclude that the main explanation of the RHI is related to changes in multisensory bodily perception, which allows room for individual variability based on a whole range of personality factors related to cognitive and perceptual processing, including hypnotisability.

## Data Availability Statement

The original contributions presented in the study are included in the article/Supplementary Material, further inquiries can be directed to the corresponding authors.

## Ethics Statement

Approval for the study on which this article is based was received from the University of Sussex ethics committee and participants gave informed consent for the study.

## Author Contributions

MS conceived the study, performed all statistical analyses, and wrote the first draft. HE contributed to the writing of the manuscript, in particular to the Sections “Introduction” and “Discussion.” Both authors developed the additional analyses involving expectancy ratings and model comparisons and approved the final version of the manuscript.

## Conflict of Interest

The authors declare that the research was conducted in the absence of any commercial or financial relationships that could be construed as a potential conflict of interest.

## Publisher’s Note

All claims expressed in this article are solely those of the authors and do not necessarily represent those of their affiliated organizations, or those of the publisher, the editors and the reviewers. Any product that may be evaluated in this article, or claim that may be made by its manufacturer, is not guaranteed or endorsed by the publisher.

## Footnotes

1
https://osf.io/7nbky/

2
https://mc-stan.org/users/interfaces/rstan

3 These results are very slightly different from those reported in Lush et al. since here we are using all 353 data points, while the degrees of freedom reported by Lush et al. indicate that they used 4 less data points. All our degrees of freedom are 351.

4
https://mc-stan.org/
