# Supplementary material for: Multisensory Integration Dominates Hypnotisability and Expectations in the Rubber Hand Illusion
Source: Front Hum Neurosci. 2022 Jun 16;16:834492. doi: 10.3389/fnhum.2022.834492 (PMC9244625; doi:10.3389/fnhum.2022.834492)
Supplement: Supplementary file 1 [file Data_Sheet_1.pdf]

Multisensory Integration Dominates Hypnotisability and Expectations in the Rubber Hand Illusion

Mel Slater and H. Henrik Ehrsson

Supplementary Material

1. Supplementary Text S1 – The Stan programs

The programs used to generate the results are available through the Kaggle system:

https://www.kaggle.com/melslater/multisensory-integration-in-the-rhi

If the reader would like to execute the code then register with Kaggle and then select the 'Copy and Edit' button at the top right of this web page. Each block of code can then be executed by clicking the small triangle on the left of each rectangular block of code. Note that on the Edit page the data itself can be examined through the Data section at the top right hand side.

2. Supplementary Text S2 - normal distribution analysis

Here, we carry out a Bayesian analysis but use a normal distribution for the likelihood for *ss* and *sa* rather than the Beta distribution used in the main manuscript. This is the Bayesian equivalent of the (Lush et al., 2020) analysis.

The model is as follows:

Likelihood:

$$ss \sim normal(\beta_{\mathrm{ss},0} + \beta_{\mathrm{ss},1} swash, \sigma_{\mathrm{ss}})$$
$$sa \sim normal(\beta_{\mathrm{sa},0} + \beta_{\mathrm{sa},1} swash, \sigma_{\mathrm{sa}})$$

$$dpdsync \sim normal(\beta_{pds,0} + \beta_{\mathrm{pds},1} swash, \sigma_{\mathrm{pds}})$$
$$dpdasync \sim normal(\beta_{\mathrm{pas},0} + \beta_{\mathrm{pas},1} swash, \sigma_{\mathrm{pas}})$$

These express the fact that the response variables each have a normal distribution with a mean equal to the linear expression in *swash*. The second parameters are the standard deviations.

Priors:

$$\text{All } \beta \sim normal(mean = 0, standard\ deviation = 10)$$
$$\text{all } \sigma \sim Gammal(2,0.1)$$

Summaries of the posterior distributions are shown in Table S1. The results are very similar to Tables 2 and 3 in the main manuscript, and the same conclusions are reached. However, the fit of this model is poor and therefore of limited predictive capability, as shown in Figure S1.

Supplementary Table S1. Summaries of the posterior distributions of the normal model showing the means, standard deviations and 95% credible intervals. Prob > 0 contains the posterior probabilities of the parameter being positive.

Parameter	Mean	SD	2.5%	97.5%	Prob > 0
Synchronous ss					
$\beta_{ss,0}$	0.33	0.18	-0.03	0.69	0.966
$\beta_{ss,1}$	0.58	0.10	0.37	0.78	1.000
σ_{ss}	1.49	0.06	1.38	1.60	
Asynchronous sa					
$\beta_{sa,0}$	-1.47	0.20	-1.87	-1.09	0.000
$\beta_{sa,1}$	0.68	0.11	0.47	0.90	1.000
σ_{sa}	1.61	0.06	1.49	1.74	
Synchronous dpdsync					
$\beta_{pds,0}$	0.17	0.38	-0.58	0.90	0.674
$\beta_{pds,1}$	0.54	0.21	0.12	0.96	0.995
σ_{pds}	3.01	0.11	2.80	3.25	
Asynchronous dpdasync					
$\beta_{pas,0}$	0.25	0.35	-0.44	0.94	0.769
$\beta_{pas,1}$	0.15	0.20	-0.24	0.54	0.772
σ_{pas}	2.74	0.11	2.54	2.96	

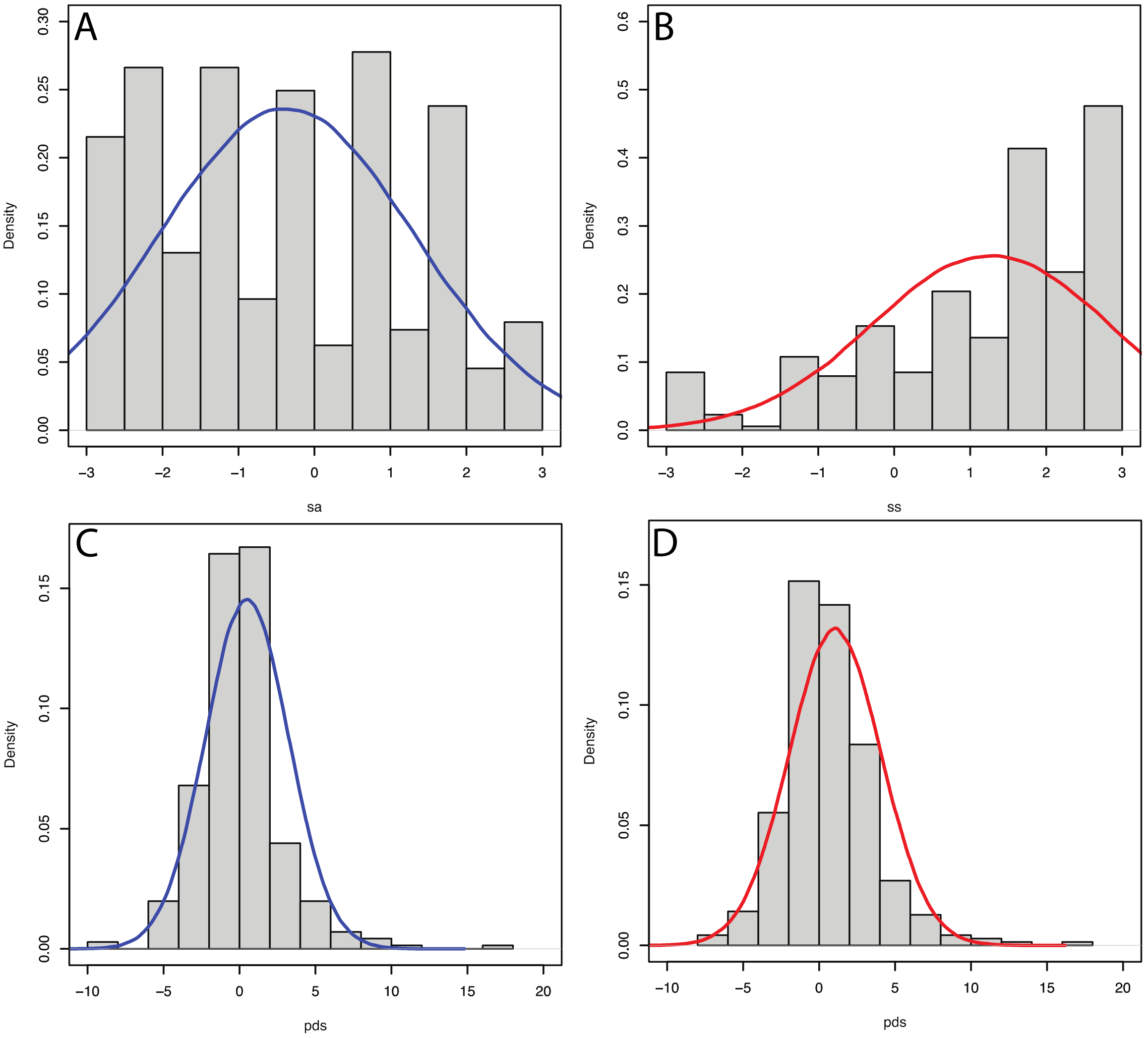

Supplementary Figure S1. Posterior predicted distributions and histograms of the observed data (A) Asynchronous questionnaire scores (*sa*), (B) synchronous questionnaire scores (*ss*), (C) asynchronous proprioceptive drift (*dpdasync*), and (D) synchronous proprioceptive drift (*dpdsync*).

3. Supplementary Text S3 – Comparing SWASH across randomly selected groups

The random variable *swash* will have a probability distribution with a certain population mean (μ) and standard deviation (σ). (In the sample of n = 353, the mean is 1.624, and standard deviation is s = 0.753). Suppose we draw a sample of n independent observations on *swash* and find the sample mean ($\bar{x}_1$). For $n \geq 30$, by the Central Limit Theorem, $\bar{x}_1 \sim$ normal$(\mu, \sigma/\sqrt{n})$. Suppose we independently draw another random sample of n independent observations on *swash* and take the mean ($\bar{x}_2$). Then similarly, $\bar{x}_2 \sim$normal$(\mu, \sigma/\sqrt{n})$. Then the difference $z = \bar{x}_1 - \bar{x}_2 \sim normal\left(0, \sigma\sqrt{\frac{2}{n}}\right)$. Now we want the probability that the absolute value of the difference between the two means (z) is at least 1. If we take sample sizes of $n = 30$ and assume that the population standard deviation σ is the same as the observed s, then $P(|z| > 1) = 2.7 \times 10^{-7}$. Similarly $P(|z| > 0.5) = 0.010$.

4. Supplementary Text S4 – Discussion

4.1 The Lush Collabra Psychology study

One of Lush's key arguments for his view that the RHI is confounded with demand characteristics is based on the results presented from a single online study conducted with 20 participants (Lush, 2020). However, this study has severe limitations that make the results difficult to interpret, as we will discuss below. In (Lush, 2020), the participants, who were most likely psychology undergraduate students because they were all compensated with course credit, had the task of guessing what participants were expected to experience in the RHI experiment with synchronous and asynchronous conditions. To learn about the RHI, the participants read texts and watched videos on their own computers about the conditions, procedures, and purpose of the experiment. They were then given rubber hand illusion questionnaires and asked to fill them out according to their expectations about what the procedures were supposed to cause. The results show that the participants rated the illusion statements significantly higher in the synchronous condition than in the asynchronous condition and significantly higher than the control statements.

The first major limitation of the study relates to the study material. The material included detailed information on synchronous and asynchronous stimulation conditions, videos showing close-ups on how the hidden right hand is touched and emphasizing the difference between the synchronous and asynchronous conditions, and information stating that the purpose of the rubber hand illusion is to 'generate changes in experience' (Lush, 2019). Moreover, the words 'synchronous' and 'asynchronous' were repeated seven times, which could have primed the participants. Notably, this information is far more extensive and detailed than the minimal information about the procedures that is typically given to participants taking part in a typical rubber hand illusion experiment who are never told anything about conditions, patterns of stroking or the specific purpose. Thus, the participants might have used the detailed information they received in combination with the information obtained from the questionnaire statements and their psychology undergraduate training to try to infer what people might be experiencing in the rubber hand illusion, which would be perfectly in line with the task instructions. The problem is that this may tell us nothing about how genuinely naïve participants might spontaneously develop expectations in real RHI experiments. Thus, Lush's study could be considered to be not ecologically valid, and it is unclear whether the results are generalizable.

The second major limitation is that the participants were never tested on the rubber hand illusion; therefore, we do not know how their 'expectations' may have modulated their subjective reports when they were exposed to real synchronous and asynchronous conditions. Although there may be a modulation, we know that perceptual illusions are robust to conceptual knowledge and that participants can experience such illusions regardless of what they think they will experience or what they are told about the illusion beforehand. Thus, it is likely that if they were exposed to the actual illusion and perceived it vividly in one condition and not in the other, most participants would simply fill out the questionnaires according to these experiences, regardless of what they may or may not have theorized would happen before the experiment commenced. Thus, expectations, even if they exist in a particular study, may only have a minor impact on the subjective reports of the RHI. Indeed, this is what the current results suggest; i.e., expectancy ratings have a tiny impact on subjective illusion ratings compared to that of the multisensory condition.

Third, it is somewhat unclear what the reported 'expectations' in Lush's study correspond to and whether the participants actually developed a precise understanding of the RHI. Notably, the participants rated their expectancies on the illusion statements in the asynchronous condition surprisingly highly, with a mean positive rating (+0.4); this indicates that the participants thought that they would feel the illusion, which is qualitatively different from the clearly negative ratings that indicate illusion denial that are given by participants after the asynchronous condition experience in real experiments (typically -2 to -3) (Kalckert and Ehrsson, 2014; Reader et al., 2021). This observation suggests that the participants probably did not fully understand the key differences in the illusion experience between the synchronous and asynchronous conditions. This interpretation is underscored by the results reported in (Reader, 2021) that used the same procedures and participant study material as those used in Lush's original study (with some small modifications). This study found that if participants were asked to describe both freely and verbally what they believed they would experience if they were a participant in an RHI experiment, the vast majority of the participants could not describe any of the typical sensations associated with the illusion (in either condition). Thus, most of the participants did not know what to expect. Collectively, the limitations discussed above mean that the results of (Lush, 2020) should be interpreted with caution and that this study cannot be used to make strong and generalizing statements about demand characteristics and expectations in RHI studies.

Note that the same problems and limitations apply to Lush and colleagues' recent follow-up study (Lush et al., 2021), in which they used the same survey approach and study material about the RHI as those used in (Lush, 2020) to examine whether psychology students can guess proprioceptive drift and threat-evoked SCR responses after receiving detailed information about these procedures. An additional problem in this latest study is that the participants' metacognitive estimations and guesses are very different from actual physiological registrations of threat-evoked SCR or manual pointing responses made during real proprioceptive drift assessments. Thus, this study tells us little about the potential influence of expectancies on indirect RHI measures.

4.2 Psychophysics, behavioural, and neuroimaging evidence against demand characteristics

Subjective report results that are difficult to explain by demand characteristics have been obtained from experimental paradigms using fine-grained stepwise manipulation of the degree of multisensory incongruence. For example, the degree of asynchrony varies around the temporal window of integration that is critical for illusion elicitation (200-300 ms) (Shimada et al., 2009); the distance between the rubber hand and the real hand systematically varies around the maximum critical distance that determines illusion elicitation according to the spatial principle of integration (and the extent of peripersonal space around the real hand) (Lloyd, 2007; Makin et al., 2008; Brozzoli et al., 2012; Kalckert and Ehrsson, 2014); the degree of relative orientation incongruence of the rubber hand and the real hand varies across angular disparities that are either tolerated or break the illusion (Ide, 2013); or the degree of humanoid shape of the fake hand systematically varies to identify the critical appearance that determines the illusion (Tsakiris et al., 2010). In these experiments, it is difficult for the participants to guess the hypothesis or precise degree of temporal or spatial incongruence that reduces the illusion.

This approach of using the subtle stepwise manipulation of multisensory incongruence has also been used in psychophysics experiments and is a rigorous method for quantifying perception at the level of single participants (Chancel and Ehrsson, 2020; Chancel et al., 2021a; Chancel et al., 2021b). For example, Chancel and Ehrsson (2020) used a two-alternative forced-choice RHI discrimination task where participants had to choose which of two rubber hands felt most like their own. In this paradigm, robots touched the rubber hands with varying degrees of relative asynchrony relative to the strokes delivered to the hidden real hand. In each trial, one of the rubber hands was touched synchronously for 12 seconds to induce an illusion, while the other rubber hand was touched for the same period with one of seven levels of asynchrony (-200 ms, -100 ms, -50 ms, 0 ms, +50 ms, +100 ms, + 200 ms) that was randomly varied from trial to trial. The result is a characteristic psychometric curve that fits a cumulative Gaussian function and describes a systematic relationship between the degree of asynchrony and the probability of choosing the asynchronously stimulated rubber hand. Notably, this finding was observed at the individual level based on data from a large number of trials that varied in illusion strength and temporal incongruence in a setup using automatic stimulus delivery with robots and with the experimenter out of view. Moreover, most participants were not even aware of how many asynchrony intervals were used, and they developed no understanding of the study’s hypotheses, as evident from post-experimental interviews. In addition, between sessions, Chancel and Ehrsson (2020) manipulated slightly the spatial relationship between the two rubber hands (one rubber hand moved 5 cm away from the other rubber hand and thus also farther away from the real hand) without the participants noticing this (as confirmed in post-experimental interviews); this manipulation produced the predicted effects on the discriminations, which were in line with the multisensory theory that reducing visuo-proprioceptive disparity increases the illusion. In our opinion, it is virtually impossible to explain these psychophysical findings by demand characteristics (or hypnotisability).

Another psychophysical finding that demand characteristics can probably not explain is the effect of introducing visual noise—thereby reducing the reliability of visual information—on RHI detection responses. According to the multisensory theoretical framework, increasing sensory uncertainty should lead to longer temporal asynchronies being tolerated when eliciting the RHI. Such sensory uncertainty effects on the RHI can be formally modelled using causal inference models of multisensory integration (Körding et al., 2007; Kilteni et al., 2015; Samad et al., 2015; Chancel et al., 2021a). In a recent preprint, Chancel et al. (2021a) quantitatively demonstrated that RHI detection judgements (i.e., reporting whether the illusion was present or not) across seven degrees of asynchrony fit the causal inference model well under conditions of varying sensory uncertainty and better so than a simpler ‘fixed criteria’ model. It is impossible that the vast majority of naïve participants in Chancel's experiment (who were not psychology students) could spontaneously develop an understanding of the quantitative predictions of the Bayesian causal inference model and generate the required detection responses across more than one hundred trials with subtle and barely noticeable differences in asynchrony in order to ‘fake’ results as implied by Lush and colleagues’ suggestions about demand characteristics. It is worth noting that the experimenter was always out-of-sight and blind to the visual noise condition. In these experiments, the participants did not even notice how many different levels of asynchrony were used in the trials (seven); most participants answered ‘four’ when they were asked to guess the number of levels in post-experimental questionnaires.

We should also say something about the objective indirect tests of the RHI, given Lush and colleagues’ critique of the usefulness of such approaches. In RHI studies, it is common practice to complement the results from subjective reports with objective tests such as proprioceptive

drift, the cross-modal congruence task, threat-evoked SRC, fMRI and other tests, as we described in the Introduction. Such objective tests provide important complementary evidence for the RHI. According to the multisensory bodily illusion account, these more objective measures are not unrelated to subjective illusion reports and should not be disregarded from the discussion simply because they do not probe subjective experience directly, as Lush and colleagues suggest. In the bodily illusion framework, the RHI corresponds to a change in the central multisensory representation of the hand, and this is associated both with changes in immediate bodily awareness, which can be quantified with subjective reports and psychophysics, as well as with changes in body representation that produce behavioural, physiological, and neural effects that can be registered with indirect objective methods. Although the causal relationship between subjective experience and the various objective measures is not fully understood and continues to be an area of research (Rohde et al., 2011; Brozzoli et al., 2012; Abdulkarim and Ehrsson, 2016; Abdulkarim et al., 2021), the condition-specific changes in proprioceptive drift, threat-evoked SCR, cross-modal congruence tasks, and fMRI activations corroborate the findings from subjective report findings very well. The objective measures show the same condition specificity and follow similar spatial, temporal and other multisensory congruence principles as those followed by the subjective reports (Lloyd, 2007; Shimada et al., 2009; Tsakiris et al., 2010; Gentile et al., 2013; Kalckert and Ehrsson, 2014; Kilteni and Ehrsson, 2017; Fang et al., 2019). Moreover, precisely because these measures are indirect and involve nonverbal behavioural and physiological changes, they are considered to be more protected against demand characteristics and high-level cognitive bias than subjective reports. Lush cites studies that have reported that hypnosis can influence nonverbal behaviour and electrodermal activity (Lush, 2020) but fails to recognize that such effects are rather unspecific and that the objective RHI findings come from comparisons with multiple well-matched control conditions and control stimuli in naïve participants that are much more difficult to explain away with demand characteristics.

Let us look at two concrete examples in more detail. Kilteni and Ehrsson (2017) measured the attenuation of perceived forces during bimanual self-touch using a well-established psychophysical procedure. They showed that the rubber hand illusion modulates tactile attenuation effects similar to a real hand across different conditions when the distance between the real hands or the distance between a rubber hand and a real hand is manipulated. The attenuation of touch only arises when the real hands are close, as in direct physical contact; the left index finger receives the force (by a robot), and the right hand, which is positioned on top of the left hand, generates the force by pressing against a force sensor that is placed directly above the left index finger. Now, when a rubber right hand is placed 25 cm to the right of the real right hand, and the real right hand is occluded, and the RHI is induced, this condition abolishes the attenuation effect as if the rubber hand is represented as the real one by the sensorimotor system. Conversely, when the (occluded) real right hand is placed 25 cm to the right of the real left hand – a spatial configuration that normally cancels attenuation – while the rubber hand is placed directly on top of the left hand during the illusion, the attenuation effect is again observed; this shows that the rubber hand is represented as the participant's own. Moreover, the stronger are the changes in subjectively reported RHI (based on a difference score that is unrelated to SWASH; (Ehrsson et al., 2022)), the stronger are the above increases or decreases in force attenuation, depending on the spatial relationship between the right rubber hand and the real left hand. Note that the specific pattern of changes in the force perception task is completely unrelated to the RHI that the participants experience; participants just report perceived force intensities using a psychophysics procedure. How the illusion should influence judgements of force according to the relative placements of rubber hands and real hands in the different conditions probably cannot be guessed by the participants spontaneously.

A second example comes from neuroimaging. When contrasting the synchronous and asynchronous conditions (and using additional controls), the RHI is associated with the replicable fMRI activation of specific areas in the premotor cortex and the cortex lining the intraparietal sulcus (Ehrsson et al., 2004; Ehrsson et al., 2005; Brozzoli et al., 2012; Limanowski and Blankenburg, 2016; Grivaz et al., 2017; Guterstam et al., 2019) . These areas are multisensory convergence regions where visual, tactile, proprioceptive and other bodily sensory signals from the upper arm are integrated and coherent multisensory representations of the arm and hand are formed (Graziano, 1999; Graziano et al., 2000; Makin et al., 2008; Brozzoli et al., 2011; Gentile et al., 2011; Gentile et al., 2013; Fang et al., 2019). Thus, the increase in activation found in these areas when contrasting the synchronous condition with the asynchronous condition (and to additional controls) supports the multisensory hypothesis of the RHI and speaks against demand characteristics or hypnotic suggestions. Participants typically do not know which areas should be activated in particular conditions; even if they did know (for example, by reading scientific papers), this would not help them because people cannot voluntarily control the level of brain activation in specific well-localized areas in the association cortex (at least not without sophisticated neurofeedback training). It is noteworthy that the degree of illusion condition-specific activation in these multisensory areas is correlated not only with the strength of subjective illusion reports (Ehrsson et al., 2004; Ehrsson et al., 2005; Brozzoli et al., 2012; Gentile et al., 2013; González-Franco et al., 2013) but also with the degree of condition-specific increase in proprioceptive drift towards the rubber hand (Brozzoli et al., 2012) and with the degree of condition-specific increase in threat-evoked SCR directed to the illusory ‘owned’ hand (Ehrsson et al., 2007; Gentile et al., 2013). Again, these correlations fit well with the multisensory bodily illusion explanation for the RHI but not with a story based on demand characteristics or trait hypnotisability. In addition, physical threats to a rubber hand (Ehrsson et al. 2007), a virtual hand (González-Franco et al., 2013), or a stranger’s body trigger BOLD responses in areas related to pain anticipation and fear (Ehrsson et al., 2007; Guterstam et al., 2015) and electrophysiological responses in the motor cortex that correlate with the strength of the body ownership illusion (synchronous vs. asynchronous). Again, such findings are very difficult to explain away with demand characteristics but fit well with the view that the rubber hand is represented as one’s own during the rubber hand illusion; thus, physical threats to the illusory owned artificial limb/body trigger similar neural emotional and motoric defensive processes as those triggered by a threat to a real limb.

Supplementary References

Abdulkarim, Z., and Ehrsson, H.H. (2016). No causal link between changes in hand position sense and feeling of limb ownership in the rubber hand illusion. *Attention, Perception, & Psychophysics* 78**,** 707-720. doi.org/10.3758/s13414-015-1016-0

Abdulkarim, Z., Hayatou, Z., and Ehrsson, H.H. (2021). Sustained rubber hand illusion after the end of visuotactile stimulation with a similar time course for the reduction of subjective ownership and proprioceptive drift. *Experimental Brain Research* 239**,** 3471-3486. doi.org/10.1007/s00221-021-06211-8

Brozzoli, C., Gentile, G., and Ehrsson, H.H. (2012). That's near my hand! Parietal and premotor coding of hand-centered space contributes to localization and self-attribution of the hand. *J. Neurosci.* 32**,** 14573-14582. doi.org/10.1523/JNEUROSCI.2660-12.2012

Brozzoli, C., Gentile, G., Petkova, V.I., and Ehrsson, H.H. (2011). fMRI adaptation reveals a cortical mechanism for the coding of space near the hand. *J. Neurosci.* 31**,** 9023-9031. doi.org/10.1523/JNEUROSCI.1172-11.2011

Chancel, M., and Ehrsson, H.H. (2020). Which hand is mine? Discriminating body ownership perception in a two-alternative forced-choice task. *Attention, Perception, & Psychophysics* 82**,** 4058-4083. 10.3758/s13414-020-02107-x

Chancel, M., Ehrsson, H.H., and Ma, W.J. (2021a). Uncertainty-based inference of a common cause for body ownership. *OSF [Preprints]*. doi.org/10.31219/osf.io/yh2z7

Chancel, M., Hasenack, B., and Ehrsson, H.H. (2021b). Integration of predictions and afferent signals in body ownership. *Cognition* 212**,** 104722. doi.org/10.1016/j.cognition.2021.104722

Ehrsson, H.H., Fotopoulou, A., Radziun, D., Longo, M., and Tsakiris, M. (2022). No specific relationship between hypnotic suggestibility and the rubber hand illusion. *Nat Commun* 13. doi.org/10.1038/s41467-022-28177-z

Ehrsson, H.H., Holmes, N.P., and Passingham, R.E. (2005). Touching a Rubber Hand : Feeling of Body Ownership is Associated with Activity in Multisensory Brain Areas. *J. Neurosci.* 25**,** 10564 -10573. doi.org/10.1523/JNEUROSCI.0800-05.2005

Ehrsson, H.H., Spence, C., and Passingham, R.E. (2004). That's my hand! Activity in premotor cortex reflects feeling of ownership of a limb. *Science* 305**,** 875-877. 10.1126/science.1097011

Ehrsson, H.H., Wiech, K., Weiskopf, N., Dolan, R.J., and Passingham, R.E. (2007). Threatening a rubber hand that you feel is yours elicits a cortical anxiety response. *Proc. Natl. Acad. Sci. USA* 104**,** 9828-9833. 10.1073/pnas.0610011104

Fang, W., Li, J., Qi, G., Li, S., Sigman, M., and Wang, L. (2019). Statistical inference of body representation in the macaque brain. *Proc. Natl. Acad. Sci. USA* 116**,** 20151-20157. doi.org/10.1073/pnas.1902334116

Gentile, G., Guterstam, A., Brozzoli, C., and Ehrsson, H.H. (2013). Disintegration of multisensory signals from the real hand reduces default limb self-attribution: an fMRI study. *J. Neurosci.* 33**,** 13350-13366. doi.org/10.1523/JNEUROSCI.1363-13.2013

Gentile, G., Petkova, V.I., and Ehrsson, H.H. (2011). Integration of visual and tactile signals from the hand in the human brain: an fMRI study. *Journal of Neurophysiology* 105**,** 910-922. doi.org/10.1152/jn.00840.2010

González-Franco, M., Peck, T.C., Rodríguez-Fornells, A., and Slater, M. (2013). A threat to a virtual hand elicits motor cortex activation. *Experimental Brain Research* 232**,** 875-887. 10.1007/s00221-013-3800-1

Graziano, M.S. (1999). Where is my arm? The relative role of vision and proprioception in the neuronal representation of limb position. *Proc. Natl. Acad. Sci. USA* 96**,** 10418-10421. doi.org/10.1073/pnas.96.18.10418

Graziano, M.S., Cooke, D.F., and Taylor, C.S. (2000). Coding the location of the arm by sight. *Science* 290**,** 1782-1786. 10.1126/science.290.5497.1782

Grivaz, P., Blanke, O., and Serino, A. (2017). Common and distinct brain regions processing multisensory bodily signals for peripersonal space and body ownership. *Neuroimage* 147**,** 602-618. doi.org/10.1016/j.neuroimage.2016.12.052

Guterstam, A., Björnsdotter, M., Gentile, G., and Ehrsson, H.H. (2015). Posterior cingulate cortex integrates the senses of self-location and body ownership. *Current Biology* 25**,** 1416-1425. doi.org/10.1016/j.cub.2015.03.059

Guterstam, A., Collins, K.L., Cronin, J.A., Zeberg, H., Darvas, F., Weaver, K.E., Ojemann, J.G., and Ehrsson, H.H. (2019). Direct electrophysiological correlates of body ownership in human cerebral cortex. *Cerebral Cortex* 29**,** 1328-1341. doi.org/10.1093/cercor/bhy285

Ide, M. (2013). The effect of "anatomical plausibility" of hand angle on the rubber-hand illusion. *Perception* 42**,** 103-111. doi.org/10.1068/p7322

Kalckert, A., and Ehrsson, H.H. (2014). The moving rubber hand illusion revisited: Comparing movements and visuotactile stimulation to induce illusory ownership. *Consciousness and Cognition* 26**,** 117-132. doi.org/10.1016/j.concog.2014.02.003

Kilteni, K., and Ehrsson, H.H. (2017). Body ownership determines the attenuation of self-generated tactile sensations. *Proc Natl Acad Sci U S A* 114**,** 8426-8431. doi.org/10.1073/pnas.1703347114

Kilteni, K., Maselli, A., Kording, K.P., and Slater, M. (2015). Over my fake body: body ownership illusions for studying the multisensory basis of own-body perception. *Frontiers in Human Neuroscience* 9. 10.3389/fnhum.2015.00141

Körding, K.P., Beierholm, U., Ma, W.J., Quartz, S., Tenenbaum, J.B., and Shams, L. (2007). Causal inference in multisensory perception. *PLoS One* 2**,** e943. doi.org/10.1371/journal.pone.0000943

Limanowski, J., and Blankenburg, F. (2016). Integration of visual and proprioceptive limb position information in human posterior parietal, premotor, and extrastriate cortex. *J. Neurosci.* 36**,** 2582-2589. doi.org/10.1523/JNEUROSCI.3987-15.2016

Lloyd, D.M. (2007). Spatial limits on referred touch to an alien limb may reflect boundaries of visuo-tactile peripersonal space surrounding the hand. *Brain and Cognition* 64**,** 104-109. 10.1016/j.bandc.2006.09.013

Lush, P. (2019). Expectancies in the Rubber Hand Illusion. osf.io/9c8mq/

Lush, P. (2020). Demand characteristics confound the rubber hand illusion. *Collabra: Psychology* 6. doi.org/10.1525/collabra.325

Lush, P., Botan, V., Scott, R.B., Seth, A.K., Ward, J., and Dienes, Z. (2020). Trait phenomenological control predicts experience of mirror synaesthesia and the rubber hand illusion. *Nat Commun* 11**,** 1-10. doi.org/10.1038/s41467-020-18591-6

Lush, P., Seth, A., and Dienes, Z. (2021). Hypothesis awareness confounds asynchronous control conditions in indirect measures of the rubber hand illusion. *Royal Society Open Science* 8**,** 210911. 10.1098/rsos.210911

Makin, T.R., Holmes, N.P., and Ehrsson, H.H. (2008). On the other hand : Dummy hands and peripersonal space. *Behavioural Brain Research* 191**,** 1-10. 10.1016/j.bbr.2008.02.041

Reader, A.T. (2021). What do participants expect to experience in the rubber hand illusion? A conceptual replication of Lush (2020). *OSF [Preprints].* doi.org/10.31234/osf.io/d8x9y

Reader, A.T., Trifonova, V.S., and Ehrsson, H.H. (2021). The relationship between referral of touch and the feeling of ownership in the rubber hand illusion. *Frontiers in Psychology* 12. 10.3389/fpsyg.2021.629590

Rohde, M., Di Luca, M., and Ernst, M.O. (2011). The Rubber Hand Illusion: Feeling of ownership and proprioceptive drift do not go hand in hand. *PLoS One* 6**,** e21659. doi.org/10.1371/journal.pone.0021659

Samad, M., Chung, A.J., and Shams, L. (2015). Perception of body ownership is driven by Bayesian sensory inference. *PLoS One* 10**,** e0117178. doi.org/10.1371/journal.pone.0117178

Shimada, S., Fukuda, K., and Hiraki, K. (2009). Rubber hand illusion under delayed visual feedback. *PLoS One* 4**,** e6185. doi.org/10.1371/journal.pone.0006185

Tsakiris, M., Carpenter, L., James, D., and Fotopoulou, A. (2010). Hands only illusion: multisensory integration elicits sense of ownership for body parts but not for non-corporeal objects. *Experimental Brain Research* 204**,** 343-352. 10.1007/s00221-009-2039-3
